# Evidence for a single, ancient origin of a genus-wide alternative life history strategy

**DOI:** 10.1126/sciadv.abq3713

**Published:** 2023-03-22

**Authors:** Kalle Tunström, Alyssa Woronik, Joseph J. Hanly, Pasi Rastas, Anton Chichvarkhin, Andrew D. Warren, Akito Y. Kawahara, Sean D. Schoville, Vincent Ficarrotta, Adam H. Porter, Ward B. Watt, Arnaud Martin, Christopher W. Wheat

**Affiliations:** ^1^ Department of Zoology, Stockholm University, Stockholm, Sweden.; ^2^ Department of Biology, Sacred Heart University, Fairfield, CT, USA.; ^3^ Department of Biological Sciences, The George Washington University, Washington, DC, USA.; ^4^ Institute of Biotechnology, University of Helsinki, 00014 Helsinki, Finland.; ^5^ National Scientific Center of Marine Biology, Far Eastern Branch of Russian Academy of Sciences, Palchevskogo 17, Vladivostok 690022, Russia.; ^6^ McGuire Center for Lepidoptera and Biodiversity, Florida Museum of Natural History, University of Florida, Gainesville, FL 32611, USA.; ^7^ Department of Entomology, University of Wisconsin-Madison, Madison, WI, USA.; ^8^ Department of Biology, University of Massachusetts Amherst, Amherst, MA 01003, USA.; ^9^ Department of Biology, University of South Carolina, Columbia, SC 29208, USA.; ^10^ Rocky Mountain Biological Laboratory, Crested Butte, CO 81224, USA.

## Abstract

Understanding the evolutionary origins and factors maintaining alternative life history strategies (ALHS) within species is a major goal of evolutionary research. While alternative alleles causing discrete ALHS are expected to purge or fix over time, one-third of the ~90 species of *Colias* butterflies are polymorphic for a female-limited ALHS called Alba. Whether Alba arose once, evolved in parallel, or has been exchanged among taxa is currently unknown. Using comparative genome-wide association study (GWAS) and population genomic analyses, we placed the genetic basis of Alba in time-calibrated phylogenomic framework, revealing that Alba evolved once near the base of the genus and has been subsequently maintained via introgression and balancing selection. CRISPR-Cas9 mutagenesis was then used to verify a putative cis-regulatory region of Alba, which we identified using phylogenetic foot printing. We hypothesize that this cis-regulatory region acts as a modular enhancer for the induction of the Alba ALHS, which has likely facilitated its long evolutionary persistence.

## Graphical abstract

**Figure:**
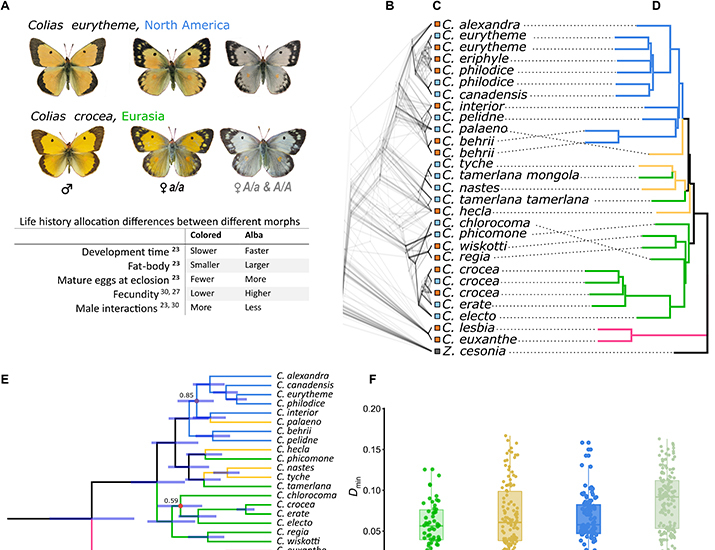

## Teaser

An ancient, trans-specific polymorphism provides insights into the evolution of life history strategies.

## INTRODUCTION

Species vary in their phenotypes, allocating resources in differing amounts to growth, maintenance, and reproduction in an attempt to maximize fitness (*1*). Across the tree of life, individuals within species also vary in these life history traits, whether plastically, genetically, or through a combination of both, due to trade-offs in allocated resources (*2*). When genetically regulated and causing distinct phenotypes, these intraspecific polymorphisms are called alternative life history strategies (ALHS) (*3*). Examining why some species remain polymorphic, as opposed to becoming fixed for one strategy, has attracted the attention of empiricists and theoreticians for decades. While theory and empirical work suggest that environmental stability has a large role in the maintenance of ALHS within populations (*4*), mechanistic insights are needed to advance our understanding of how these polymorphisms affect eco-evolutionary dynamics during adaptive diversification of populations and species (*4*, *5*). Unfortunately, the genetic basis of ALHS remains poorly understood outside of laboratory species, limiting our understanding of how life history traits evolve (*2*).

When a new genetically determined ALHS arises in a population, directional selection is predicted to eventually purge or fix this variant over time (*6*, *7*). Thus, maintenance of an ALHS in multiple closely related species (such as within a genus) indicates that at least one of the following about the ALHS must be true: (i) It arose once and has been maintained by some form of balancing selection (*8*), (ii) it evolved convergently in separate species (*9*, *10*), or (iii) it moved between species via introgression (*11*, *12*). In butterflies, the best genotype to phenotype connections have been made in studies of mimicry phenotypes, and these systems provide evidence for two of these alternative scenarios. Evolution via balancing selection and allelic turnover at a single locus is seen among the mimicry phenotypes of *Papilio* species, where there is little evidence of a role for introgression (*13*). In contrast, adaptive introgression of color pattern alleles is common among *Heliconius* species (*14*–*16*), although the fixation of these alleles in new genomic backgrounds involves extensive compensatory evolution to resolve negative pleiotropic effects (*17*–*19*). Given that ALHS have more diverse phenotypic impacts than wing pattern mimicry alleles, ALHS alleles might be expected to have even more negative pleiotropic effects in novel genomic backgrounds and hence little reticulated evolutionary history. Here, we seek to address these issues and advance eco-evolutionary studies by investigating the origins and evolutionary dynamics acting upon an ALHS.

**Fig. 1.**
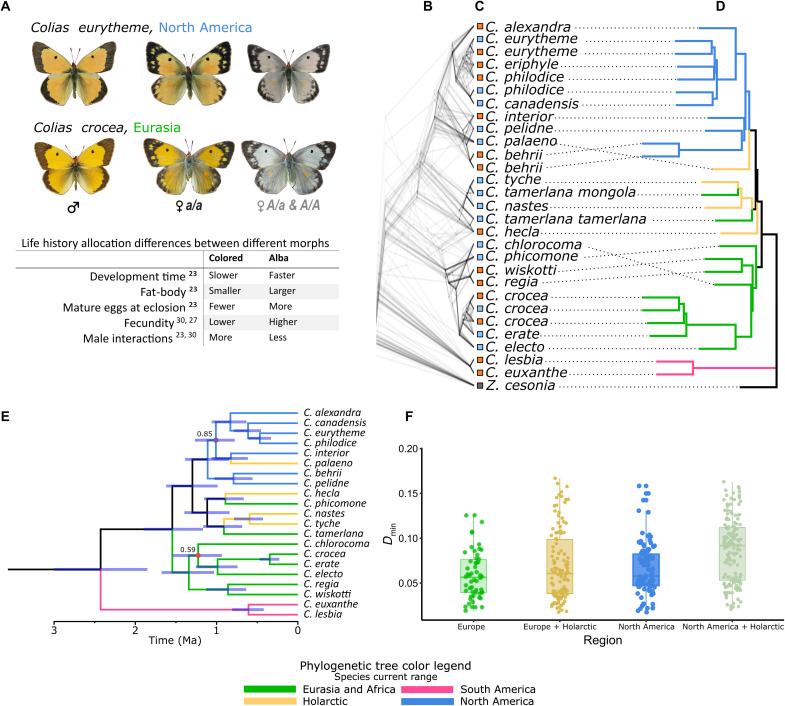
The Alba phenotypes of representative *Colias* species, the evolutionary relationships among major *Colias* lineages in light of their Alba phenotypes and regional distribution, and evidence for historical introgression. (**A**) Representative *Colias* species from both sides of the Atlantic, illustrating the female-limited Alba phenotype along with a table of life history differences between the female morphs. (**B**) A densitree plot of chromosome-level trees (one tree per chromosome), generated using gene trees based on a single exon per single-copy gene (on average 129 genes per chromosome; *n* = 4011 BUSCO genes). (**C**) Each specimen’s wing color is indicated with colored boxes on branch tips (blue = Alba, orange = colored, gray = NA). (**D**) ASTRAL species tree, generated using the longest exon per BUSCO gene, with branches color-coded by their sample’s regional distribution (blue = North America, orange = Holarctic, green = Eurasia and Africa, purple = South America). (**E**) Time-calibrated SNAPP tree generated using a subset of taxa and 1314 SNPs, with millions of years on the *x* axis. Blue bars at nodes represent 95% highest posterior distribution of node ages, with nodes having posterior support of <0.9 indicated with a dot and their value. (**F**) Distribution of minimal D-statistic of all species trios that showed significant levels of introgression (Bonferroni-Holm–corrected *P* < 0.05). Trios are grouped by Eurasian or North American regions and their respective combinations with the Holarctic taxa.

Butterflies in the genus *Colias* are characterized by their yellow, orange, or red wing coloration. However, at least a third of the approximately 90 *Colias* species have a female-limited ALHS called Alba, where female wings are white (Fig. 1A) (*20*, *21*). The remaining species are fixed for either Alba or colored wings. Alba females exhibit white wings because they have reallocated metabolic resources from wing pigmentation to reproductive development (*22*–*27*). This reallocation of resources results in a trade-off where Alba females gain an increased lipid reserve, faster development rate, and increased fecundity compared to orange females. Genetic studies in six *Colias* species consistently found that Alba is caused by a single dominant, autosomal locus (*20*). In *Colias crocea*, a Eurasian species polymorphic for the Alba ALHS, the *Alba* locus has been mapped to a transposable element insertion downstream of the gene *BarH1* (*28*). *BarH1* encodes a transcription factor (TF), and the insertion is associated with a gain of *BarH1* function in the developing wings of Alba females. This morph-specific expression difference results in a decrease in the number of pteridine pigment granules in Alba wing scales (*28*). These findings corroborated earlier observations of reduced pteridine pigments in Alba wings compared to colored wings (*22*). Despite the advantages that Alba females gain from this resource trade-off, Alba remains polymorphic, rather than proceeding to fixation, in many populations and species. A range of biotic and abiotic factors contribute to the maintenance of Alba’s intermediary frequency (*20*). Differences in the relative fitness of each morph have been attributed to temperature (*29*), host plant quality (*23*), interspecific interactions with other white pierids (*30*), and male harassment (*23*). While it has been suggested that Alba is a homologous, potentially an orthologous trait within *Colias*, the origin of Alba remains unknown outside of a single species (*20*, *21*).

To resolve this issue of orthology in an ALHS across taxa, we focus upon two distantly related species in which the Alba life history trade-off between color production and reproductive investment has been studied in detail: the North American *C. eurytheme* (*23*) and Eurasian *C. crocea* (Fig. 1A) (*24*, *26*). These species are members of two of the three most divergent and diverse clades of *Colias* (*31*). Thus, if Alba is orthologous in these two species, then the genus *Colias* can be used as a model system for studying the evolutionary dynamics of an ALHS in different genetic backgrounds and divergent ecological contexts.

Here, we investigate the evolutionary origins of an ALHS and the dynamics that have maintained it across a species-rich genus of butterflies. Specifically, we test for the alternative (single or multiple origins) and complementary evolutionary mechanisms (balancing selection and introgression) responsible for the prevalence of the Alba ALHS across *Colias*. Using a combination of phylogenetic analyses, a genome-wide association study (GWAS), and genetic manipulation, we find that (i) the Alba polymorphism arose once, likely at the root of the genus, and (ii) introgression has occurred many times during the formation of the genus, with both introgression and balancing selection recently affecting the *Alba* allele and (iii) we identify a narrow region within the *Alba* locus that likely acts as a cis-regulatory enhancer to control this trans-specific ALHS.

## RESULTS

### Phylogenomic analysis

We first reconstructed the phylogeny of *Colias* as a comparative framework for our functional genomic work. We generated a chromosome-level genome assembly and annotation for *C. eurytheme* (table S1 and figs. S1 and S3). We then aligned reads from 21 individually sequenced *Colias* species [one to three samples per species, one to two locations per species (Fig. 1, table S2, and fig. S23); for three species (*C. crocea, C. eurytheme*, and *C. philodice*), both morphs were sampled] to this reference genome and then mined each species gene set of single-copy orthologs. Many of the selected species are from North America and Europe, although the global distribution of *Colias* is represented (fig. S23 and table S1). In addition, species from both sides of the Atlantic varied in whether females are polymorphic, fixed for, or without the Alba morph. We then used the longest exon per gene, for single-copy ortholog genes (BUSCO), to estimate maximum-likelihood gene trees (*n* = 4011 exons), followed by species-tree estimation using ASTRAL. Although there was extensive conflict among gene trees (Fig. 1B and fig. S4), the species tree (Fig. 1C) supports three conclusions: Species from South America are sister to the remaining species of *Colias*; the major divergence within *Colias* is between a North American and a Eurasian + African clade; and Holarctic (currently circumpolar) taxa fall between and among these two major clades (Fig. 1C). To further assess these relationships in a multispecies coalescent framework and to estimate a divergence time between the North American and the Eurasian + African clades, we used a Bayesian species-tree inference approach (SNAPP) calibrated on an age estimate of when *Colias* and its sister genus *Zerene* last shared a common ancestor (*32*). Following recommendations to reduce the computational demands of SNAPP (*33*), we analyzed a random set of 1314 single-nucleotide polymorphisms (SNPs) selected from a reduced taxon set that retained regional diversity, but removed redundancy among closely related species. The resulting SNAPP phylogeny was largely concordant with the ASTRAL species tree, with strong support for nodes separating derived North American from Eurasian + African clades (Fig. 1D). The mean crown age of *Colias* was estimated at 2.43 million years old (median of the 95% highest posterior density (HPD), 3.02 to 1.83), while the mean age of the last common ancestor of the non–South American *Colias* was estimated to be 1.55 million years ago (1.90 to 1.17). Using these results, we assessed Alba and putative Alba phenotypes in a global phylogenetic context, revealing that Alba is present in all clades of *Colias*, regardless of geography (Fig. 1, B to D). Thus, despite extensive phylogenetic conflict in our analyses, *Colias* appear to have rapidly diversified into regional clades. While incomplete lineage sorting likely explains much of this conflict, introgression during this radiation is likely and potentially an important driver to propagate Alba among species and regions.

### Genome-wide introgression analysis

**Fig. 2.**
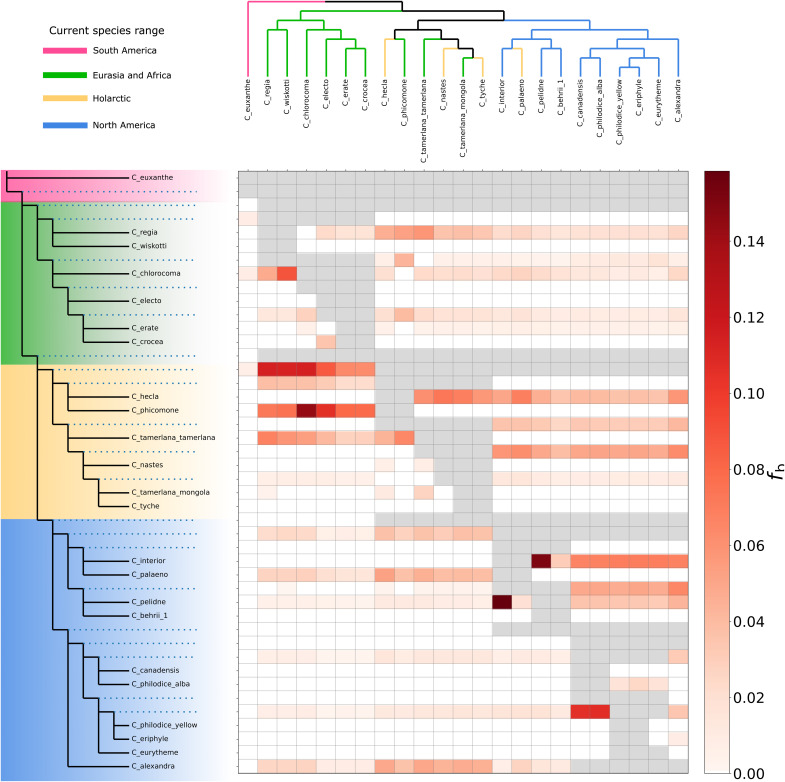
Signatures of historical introgression across a modified *Colias* species-tree phylogeny where the placement of *C. phicomone* and *C. palaeno* has been changed according to their placement in the SNAPP tree. Each cell in the grid indicates the f-branch statistic *f*_b_, identifying excess sharing of derived alleles between branch nodes on the *y* axis (blue dotted lines) and individual species on the *x* axis. A darker color in the heatmap indicates higher *f*_b_, suggesting gene flow between that branch and species. Results indicate a strong signal of introgression between *C*. *phicomone* with Eurasian taxa, as well as between an ancestor of the *C. nastes* clade and both Eurasian and North American taxa. There is also a strong signal of introgression between *C. interior* and *C. pelidne.* Species and internal nodes are colored by the species’ current distribution where purple = South America, blue = North America, orange = Holarctic, and green = Eurasia and Northern Africa.

It is well known that many *Colias* species frequently hybridize (*34*). Therefore, we expected to find substantial amounts of introgression among the species we examined. As a first step to evaluate the degree to which introgression may have shaped the phylogenetic distribution of Alba, we quantified genome-wide introgression among *Colias* species using D-statistics by analyzing all possible species trios for introgression, using the South American *C. lesbia* as an outgroup. When we grouped the resulting trios by the origin of their component species [Eurasia + Africa, Holarctic, and North America (fig. S23)], we found significant levels of introgression within these groups of non-South American species (Fig. 1F and figs. S24 and S25). In addition, the proportion of significant trios showing introgression, as well as the general level of introgression, increased when we combined taxa from the Holarctic with either North America or Eurasia + Africa groups, indicating introgression between these groups that likely happened in the past. To estimate when the introgression events among the non-South American species occurred, we integrated our ABBA-BABA analysis with our species tree and used the f-branch metric (*35*), which differentiates signatures of historical introgression between internal branch nodes from introgression between extant species. This revealed introgression between an ancestor of the Holarctic *C. hecla* + *nastes* clade (*C. nastes*, *C. tamerlana*, and *C. tyche*) and the Eurasian species and between *C. phicomone* and an ancestor of the North American species (fig. S22). We also observed significant introgression between *C. pelidne* and *C. interior*, two species that are known to hybridize (*36*). Since species in the *nastes* clade are fixed for Alba, the *Alba* allele may have been transferred through introgression between this clade and ancestors of the Eurasian species. To account for any biases in the ASTRAL species tree that we used for this f-branch analysis, we generated an additional analysis where *C. phicomone* and *C. palaeno* were placed according to their placement in the SNAPP tree (Fig. 2). Using this modified tree, we can now see that *C. phicomone* is instead only showing extensive introgression with Eurasian taxa and nothing with North American taxa. This is much more consistent with realistic biological scenarios given the current distribution of the species. However, the relocation of *C. palaeno* to be a sister species of *C. interior* did not noticeably affect estimates of introgression between *C. interior* and *C. pelidne.* However, this genome-wide analysis is unable to resolve the direction of introgression or capture localized intrachromosomal introgression events.

### Identification of the Alba locus in *C. eurytheme*

**Fig. 3.**
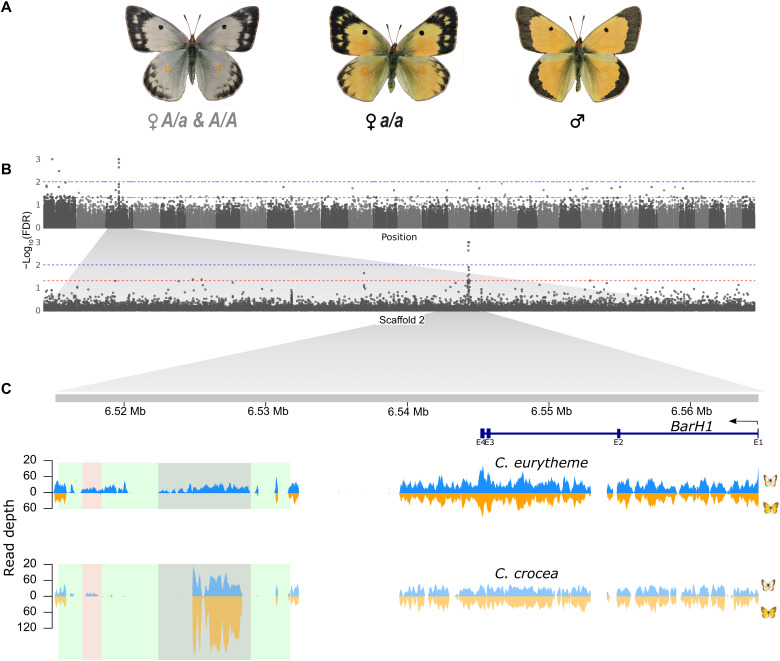
The *Alba* locus in *C. eurytheme*. (**A**) *C. eurytheme* specimens, depicting female phenotypes and genotypes. *Alba* is the dominant allele. (**B**) Results of the GWAS using data from 14 orange and 15 Alba wild-caught *C. eurytheme* females aligned against the Alba reference genome. Alternating gray-scale blocks in the Manhattan plot are colored by chromosome, ordered Z:31; the *y* axis represents negative log value Bonferroni-Holm–corrected false discovery rate [−log10(FDR_BH)] of each variant, the *x* axis is scaffold position, and the 0.05 and 0.01 percentile of the distribution is indicated with red and blue horizontal dashed lines, respectively. The second row is a close-up of scaffold 2 from chromosome 3, which contains the *Alba* locus. (**C**) Detailed view of the 58-kb region harboring the SNPs significantly associated with *Alba.* The gene model indicates the location of the BarH1 gene and its antisense reading frame (blue). Across this region, we show the read-mapping depth of whole-genome sequence data from an Alba (blue) and orange (orange) female from *C. eurytheme* (top row) and *C. crocea* (bottom row), respectively. The reads were aligned to the entire Alba *C. eurytheme* reference genome (filtered to MAPQ > 20 and proper pairs). Colored boxes highlight the *Alba* insertion (green), annotated repetitive content within the insertion (gray), and a region absent in orange individuals of both *Colias* species, which we refer to as the *Alba* candidate locus (pink). The gray region unique to Alba individuals in *C. eurytheme* is not unique to Alba individuals in *C. crocea* and is found to have elevated read depth coverage in all Eurasian species. The high variance in coverage to the left of the BarH1 locus is due to the high levels of repetitive content in this region, with reads mapping multiply and getting filtered out.

To test whether Alba has a shared or de novo origin among species, we next mapped Alba’s genetic basis in *C. eurytheme* from North America, which represents a deeply divergent lineage from European *C. crocea* where the *Alba* locus is known (*28*). Using the chromosomal linkage map from our genome assembly, we were able to associate the *Alba* locus with chromosome 3 (Fig. 3B and fig. S20B). To further narrow down the *Alba* locus, we performed an independent GWAS by mapping genomic data from 15 Alba and 14 orange wild-caught females to the reference genome. This identified two loci, the most significant of which was a single locus on chromosome 3 situated immediately downstream of the *BarH1* gene (fig. S6; but also Fig. 3C), which is concordant with both our previous mapping (fig. S20B) and the location of the *Alba* locus identified in *C. crocea* (*26*). The second locus, which had less support, was located on a different chromosome between a PIFI-like helicase and a PiggyBac transposon (fig. S7). We hypothesized that the second locus was an artifact from aligning reads to a reference genome lacking the *Alba* insertion since the reference was from an orange female individual. To test this, we generated an Alba genomic reference by combining a draft assembly made using linked read technology from an Alba *C. eurytheme* female and the reference genome (fig. S8). This added ~36 kb of sequence downstream of *BarH1* that was absent in the orange assembly. When the GWAS was repeated using this synthetic Alba reference genome, only the previous *BarH1*-associated locus remained (Fig. 3C), indicating reference bias as the likely cause of the second peak when mapping to the orange assembly reference.

### Investigating the Alba insertion

To further investigate the Alba-associated insertion region, which we expected to be composed of repeat content and regions unique to Alba, we conducted a read depth analysis by mapping the *C. eurytheme* individual genomes from the GWAS onto the Alba genome. By contrasting uniquely mapped reads of expected coverage depth against (i) reads mapping at higher-than-expected depth or (ii) reads not mapping uniquely, we could distinguish between unique *Alba* content and low complexity or repeat regions found in other parts of the genome. In the Alba-associated insertion region, we identified an ~20-kb region (Fig. 3C, green box) containing two stretches of unique *Alba* content (where no reads from orange individuals mapped; Fig. 3C, pink and gray boxes); data from orange females showed no unique content (Fig. 3C). Next, we similarly aligned reads from orange and Alba *C. crocea* females (*n* = 15 each), revealing that only one of these two regions contained reads unique to *Alba* in both species (Fig. 3). This latter region was 1200 base pairs (bp) long and had high sequence similarity between the two species, with alignment of 14 *C. eurytheme* haplotypes and 10 *C. crocea* haplotypes having 96% identity (35 fixed differences over 960 bp) (Fig. 1E). We hypothesized that this shared region causes the Alba ALHS and hereafter refer to it as the *Alba* candidate locus (Fig. 3C, pink box). We further documented that the *Alba* candidate locus was unique to Alba *C. eurytheme* individuals by assaying additional wild-caught females of each color morph with polymerase chain reaction (PCR) (*n* = 8 per morph; fig. S9).

### Comparative analysis of Alba candidate locus

To test the hypothesis that our identified *Alba* candidate locus is associated with Alba in additional *Colias* species, we generated a draft genome for *C. nastes*, a Holarctic species fixed for the Alba-color phenotype. In *C. nastes*, the *Alba* candidate locus and the *BarH1* gene assembled as a single contig, orthologous to the ones identified in *C. crocea* and *C. eurytheme*. This is consistent with the prediction that Alba has a single evolutionary origin and thus a shared ancestry among *Colias* species (figs. S10 and S11).

**Fig. 4.**
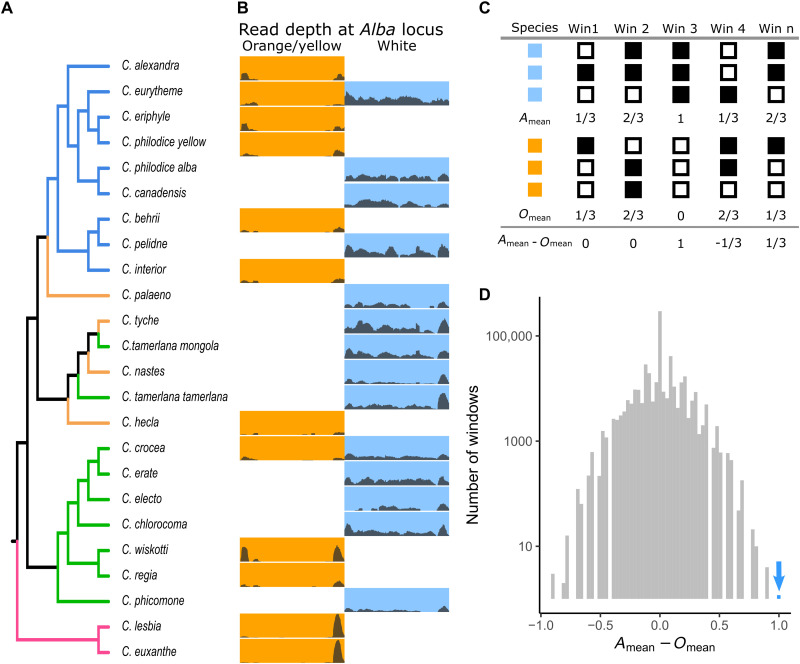
Association of the *Alba* candidate locus with wing color across the *Colias* phylogeny. (**A**) Species tree of *Colias* colored by geographic region, with purple = South America, blue = North America, orange = Holarctic, and green = Eurasia and Northern Africa. (**B**) Read-mapping depth of an individual using whole-genome data across the 1200-bp long *Alba* candidate locus for each species. Separate columns depict coverage plots according to female wing color. In cases where we have sequence data for both morphs, both are shown side by side. The *y* axis in each row is 0 to 100 × coverage. (**C**) Schematic explaining our genome-wide, window-based analysis of color-associated read coverage. Data for each species are by row, with wing color indicated by a blue or orange box. For four genomic windows, the presence of read coverage in a window is indicated by a black filled box (absence of coverage an empty box). Mean value for each window for Alba samples (*A*_mean_) and colored samples (*O*_mean_) is then calculated, followed by their difference. (**D**) Histogram showing the distribution of color-associated bias in coverage (*A*_mean_ − *O*_mean_) of 546,228 windows across the genome, with counts on the *y* axis plotted on a log_10_ scale. The one window located in the Alba candidate locus is the only one that perfectly correlates with color and is indicated with a blue bar and arrow.

We then tested the hypothesis of a shared origin of Alba by quantifying the association between having the *Alba* candidate locus and the Alba phenotype in our full species genomic dataset (*n* = 21 species; Fig. 1C). All species with white wings had mapped reads covering the entire 1200-bp-long *Alba* candidate locus except for *C. phicomone,* where reads covered approximately half the candidate locus (Fig. 4B). In contrast, none of the samples from females with colored wings (yellow, orange, or red) had reads covering the insertion region. Instead, the reads from colored wing individuals aggregated in low complexity areas flanking the locus. Thus, we observed a complete correlation between presence of the insertion (i.e., the *Alba* candidate locus) and the Alba phenotype across *Colias* species (Fig. 4). To estimate the likelihood of such a correlation on a genomic scale across species, we performed a window-based analysis of read coverage across the genome for all species (*n* = 546,228 windows, 600 bp in length each, *n* = 21 species). For each window of the genome, we then assessed read coverage for each species and quantified whether this showed any relationship with that sample’s Alba phenotype (Fig. 4C). This revealed that a window located in the *Alba* candidate locus was the only region in the entire genome, across all species, where read coverage segregated with female wing color (Fig. 4D). These findings are consistent with the *Alba* insertion causing Alba across *Colias*.

### Phylogenetic analysis of the Alba locus

**Fig. 5.**
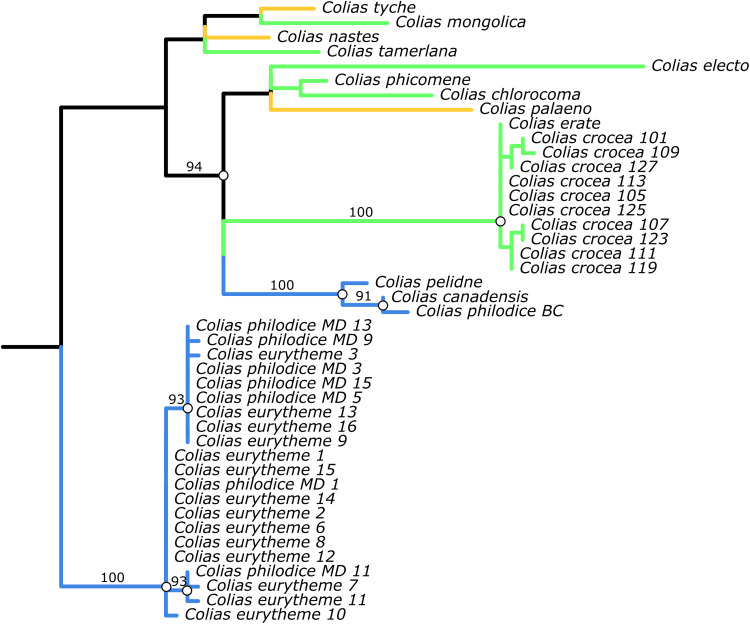
A phylogeny using only DNA data from the 1.2-kbp-long *Alba* candidate locus from Alba individuals in our species dataset, including additional samples from *C. philodice* (from Maryland, USA) and the previous GWAS study of *C. crocea* (from Cataluña, Spain). Branch color corresponds to geographic region (blue = North America, orange = Holarctic, and green = Eurasia and Northern Africa), and branch length corresponds to substitution differences. Sample clustering suggests that alleles are shared between *C. eurytheme* from California and *C. philodice* originating from Maryland (*philodice* names ending in MD). Also, note the distinct variation among alleles in both these species (there is branching within this clade). This sharing of allelic variation between species from opposite sides of North America is consistent with both the long-term maintenance and introgression of *Alba* alleles. Similar evidence of allele sharing and diversity among *Alba* alleles is seen within *C. crocea* samples, which includes an allele from *C. erate*. Nodes with support > 90% indicated by white circle and the support value upon that branch.

To further investigate the evolution of the *Alba* candidate locus, we constructed a gene tree using only data from the 1200-bp *Alba* region (Fig. 5). By comparing the resulting *Alba* gene tree with the species tree (Fig. 1), we identified instances of gene-tree species-tree concordance and discordance in species complexes on both sides of the Atlantic. In Eurasia, the multiple Alba alleles sampled from Spanish and Italian *C. crocea* form a polytomy with *C. erate* from Japan. Hybridization between these two species is well documented (*34*, *37*). This sharing of *Alba* alleles could be due to incomplete lineage sorting of *Alba* alleles in both species since they diverged, their maintenance via balancing selection in both species, the introgression of *Alba* alleles between species, or some combination of these scenarios. A similar pattern is seen in North America, where *C. eurytheme* and *C. philodice* are young sister species that actively hybridize when in contact (*38*). Despite our samples of these species coming from populations on opposite sides of North America (*C. philodice* from Maryland, *C. eurytheme* are from California), we found *Alba* alleles shared between species (Fig. 5, blue branches).

The *Alba* gene tree also revealed two instances of discordance with the species tree, suggestive of historical introgression events of the *Alba* allele. In North America, the *Alba* allele found in the *Colias philodice* collected from British Columbia forms a cluster with *C. canadensis* and *C. pelidne* (both also collected in this region) (Fig. 5). This is discordant from the species tree, which groups this *C. philodice* and *C. canadensis* with *C. eurytheme* (Fig. 1, D and E). This suggests an evolutionary history for the *Alba* allele in the clade of *C. canadensis* and *C. philodice*, independent from that found in *C. eurytheme*. Our f-branch results suggest a role for introgression between these taxa (Fig. 2). Moreover, this cluster (*C. philodice*, *C. canadensis*, and *C. pelidne*) is grouped closer to Eurasian rather than North American species in the *Alba* tree, suggesting rather divergent lineages of *Alba* alleles among North American species. In Eurasian lineages (Fig. 5, green branches), a similar discordance is seen in the *Alba* tree, where *C. electo* is placed as a distant outgroup to, rather than grouped together with, its closest sampled relatives *C. crocea* and *C. erate* (Fig. 1, D and E).

**Fig. 6.**
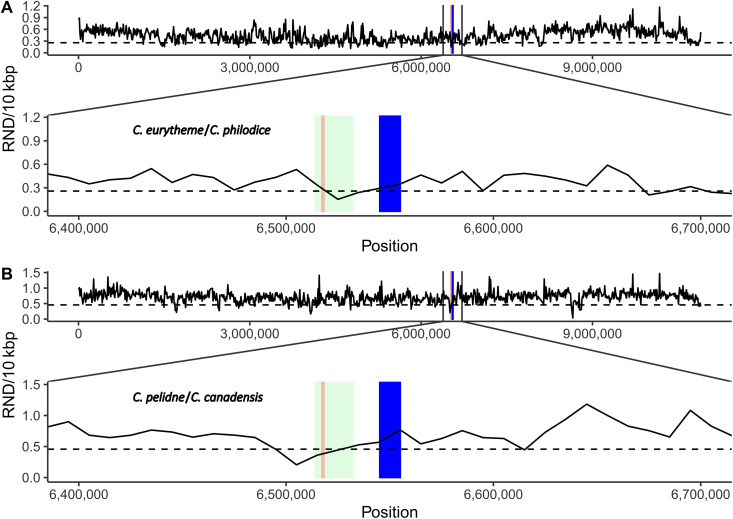
Evidence of localized introgression of the *Alba* locus. Across the genome, relative node depth (RND) is expected to show a consistent pattern of divergence between species reflecting time since their common ancestor, while RND is expected to be lower in regions of recent introgression between species. (**A**) RND between all sequenced *Alba C. eurytheme* (California, USA) and *C. philodice* (Maryland, USA) and (**B**) *C. pelidne* and *C. canadensis*. RND was estimated in nonoverlapping windows of 10 kbp along the scaffold using *C. crocea* (Eurasia) as an outgroup species. The *BarH1* gene is indicated with a blue box, and the *Alba* locus is indicated by a green and red box, as per Fig. 3C. The lower 5% of RND values is shown for scaffold 2 [length = 10.9 Mbp; dashed lines at (A) 0.258 and (B) 0.457]. Notice that while RND fluctuates across the entire scaffold in both comparisons, we observe a significant drop in RND near the *Alba* locus, but not the *BarH1* gene, consistent with a more recent shared origin of the former.

To more formally test whether *Alba* alleles have introgressed among taxa, as suggested by these patterns of discordance, we used estimates of relative node depth (RND) across the genome. RND measures genetic divergence between two species while controlling for variation in mutation rate using an outgroup species (*39*). Across the genome, RND is expected to reflect species divergence, while any introgressed regions between them should have reduced RND proportional to the time since introgression (i.e., more recent shared ancestry results in lower RND) (*39*). After estimating RND using our population samples of both *C. eurytheme* and *C. philodice Alba* individuals, we find a significant decrease in RND near the *Alba* locus, consistent with the introgression of *Alba* alleles between these taxa (Fig. 6A), as suggested by the *Alba* gene tree (Fig. 5).

We next shifted to assessing whether we can detect similar evidence of *Alba* allele introgression between more divergent taxa. *C. pelidne* and *C. canadensis* last shared a common ancestor over a million years ago (Fig. 1E), are sympatric in a large part of their range (Northwestern North America), and could potentially hybridize. While no strong signatures of genome-wide introgression were found between them in the f-branch analysis (Fig. 2), these species have a very similar *Alba* allele (Fig. 5), making them a good candidate pair to test for Alba allele introgression using RND. We observe a significant drop in RND near the *Alba* candidate locus (Fig. 6B), similar to the pattern observed between *C. eurytheme* and *C. philodice*. In sum, while our previous analyses of genome-wide introgression indicate that divergent *Colias* species have hybridized over time (Fig. 2), these patterns of discordance (Figs. 1 and 5) and the RND analyses (Fig. 6) suggest introgression of the *Alba* locus between sister species, as well as between more divergent species.

### Tests of localized introgression of Alba

**Fig. 7.**
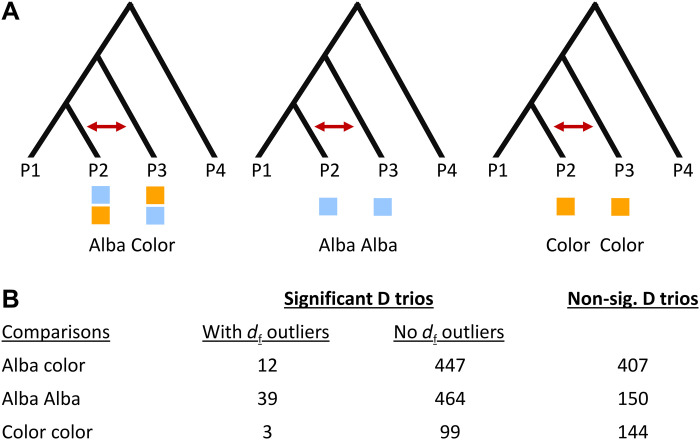
Pattern of increased localized introgression (detected using *d*_f_) at the *Alba* locus among Alba species compared to non-*Alba* species using trios binned into three groups: (i) Alba-Alba, (ii) Alba-colored, and (iii) color-color. (**A**) The sliding window analysis of introgression (*d*_f_, 500 SNP windows) was calculated for each of the trios that had significant levels of genome-wide introgression estimated by positive D (shown in Fig. 1F). These trios were then grouped according to the wing color of the introgressing species in positions P2 and P3. (**B**) Number of trios that had higher *d*_f_ across the *Alba* locus than the 95th percentile for its chromosome. Note that only Alba individuals from *C. crocea* and *C. eurytheme* were used, which deflates the number of color comparisons.

We next sought to quantify how common such *Alba* locus introgression events are in our full species dataset. Specifically, we sought to test whether there are higher levels of introgression of the *Alba* locus among the Alba species compared to colored species. To do this, we used an alternative to Patterson’s D, called distance fraction (*d*_f_), that is appropriate for window-based analysis and capable of detecting old introgression events, as it accounts for genetic distance across possible trio configurations (*40*). We calculated *d*_f_ in all unique trios in which we had previously detected significant levels of introgression using D (*n* = 1194; Fig. 1F). We then grouped results from D and *d*_f_ based on the wing color morph of the introgressing species (in positions P2 and P3; Fig. 7A). In trios with significant D, there are diverse regions of elevated *d*_f_ across chromosomes. This is also true in the chromosome containing *BarH1*, where *d*_f_ at the *Alba* locus is significantly higher than genomic background in some of these trios (fig. S5). We reasoned that if introgression has played a role in maintaining Alba in the *Colias* genus by moving it among lineages, signatures of introgression around the *Alba* locus should be more common between Alba-Alba species pairs compared to other morph pairs (since the former pairs potentially indicate successful introgression of the Alba allele). After grouping trios based on female wing color of the introgressing pairs, there were more significant trios for genome-wide estimates of introgression using D, despite there being more Alba-color comparisons (Alba-color 447 sig. trios of 866 total trios, Alba-Alba 464 sig. trios of 653 total trios, and color-color 102 sig. trios of 246 total trios). Focusing upon the *Alba* locus region using *d*_f_, there are significantly more trios with outlier values around the *Alba* locus in Alba-Alba comparisons, compared to other morph groupings (χ^2^ = 14.223, *d*_f_ = 2, *P* = 0.0008156; Fig. 7B). In sum, our detection of introgression events within the *Alba* locus region is consistent with *Alba* locus alleles having historically introgressed among lineages, potentially altering the color of the recipient lineage. However, these signatures of introgression for specific regions of the genome can be difficult to distinguish from balancing selection (*41*).

### Test for balancing selection

Although balancing selection has been invoked to explain the maintenance of Alba within populations (*30*), evidence from molecular tests of selection are lacking. Unfortunately, the indel architecture of the *Alba* locus—where the *Alba* allele either is present, large (8 to 20 kb), and highly repetitive (Fig. 3C) (*28*) or absent—makes traditional tests for balancing selection, such as Tajima’s D (*42*) and beta-statistics (*43*), unsuitable. This is because these statistics rely upon intermediate frequency SNPs in linkage disequilibrium with the causal locus; indel genotypes cannot be used.

Unfortunately, the flanking regions of the *Alba* locus have a low complexity due to high levels of repetitive content, resulting in low power to detect balancing selection at and around the *Alba* locus. Hence, we were not surprised that we could not detect any increase in Tajima’s D, nucleotide diversity, *D*_xy_, or beta-statistics surrounding the *Alba* locus when using our individual genome samples from the wild (figs. S13 and S14).

**Fig. 8.**
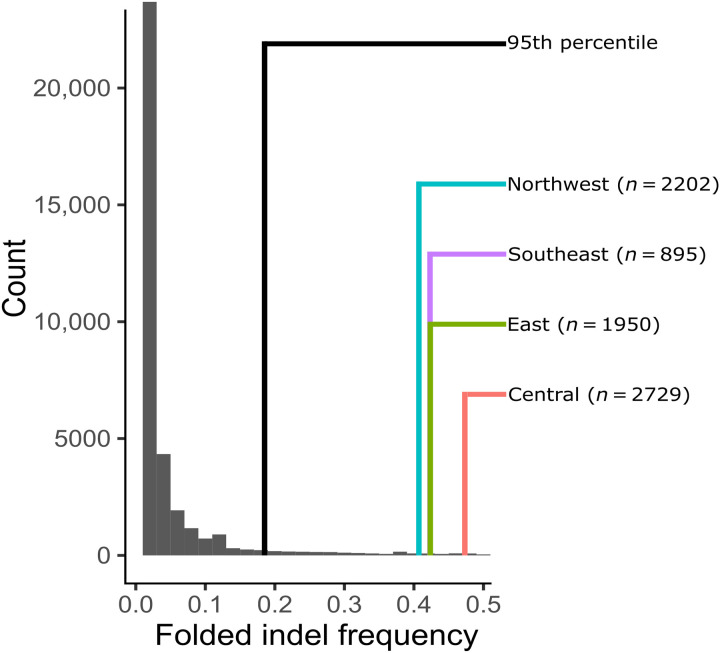
Evidence of balancing selection and introgression at the *Alba* locus. The folded allele frequency spectrum of all high-confidence insertions and deletions (*n* = 35,282 indels) identified in a field sample of 29 *C. eurytheme* females. The black line indicates the 95the percentile of this distribution. The colored lines represent the estimated allele frequency of the *Alba* allele of four different populations in California (*44*) and their sample sizes (Central population = Westley, Yarmouth, and Vernalis; Northwest population = Tracy and County Line; East population = Manteca, Rippon, and Modesto; Southeast population = Patterson and Newman). The estimated frequency of *Alba* in these field samples is more common than expected based on the genome-wide frequency of similar indels.

Instead, we developed an alternative approach comparing the frequency of Alba to the allele frequency distribution of similar derived indels across the genome in our population sample (Fig. 8). First, we mapped our field-collected GWAS samples of *C. eurytheme* against the orange *C. eurytheme* reference genome and then filtered identified structural variants for high-confidence intergenic insertions or deletions. After filtering, 35,282 structural variants remained, 95% of which occurred at a frequency of 0.185 or less (Fig. 8). On the basis of previous extensive field collections (*44*) that remain representative of populations today, 66 to 72% of females are Alba in northern California near our field site (*44*). Assuming Hardy-Weinberg proportions and *Alba* dominance, this corresponds to an *Alba* allele frequency of 0.4 to 0.47. Thus, Alba population frequencies are significantly more common than genome-wide indel frequencies in the wild and consistent with balancing selection acting upon the *Alba* locus.

### Functional validation of insertion

**Fig. 9.**
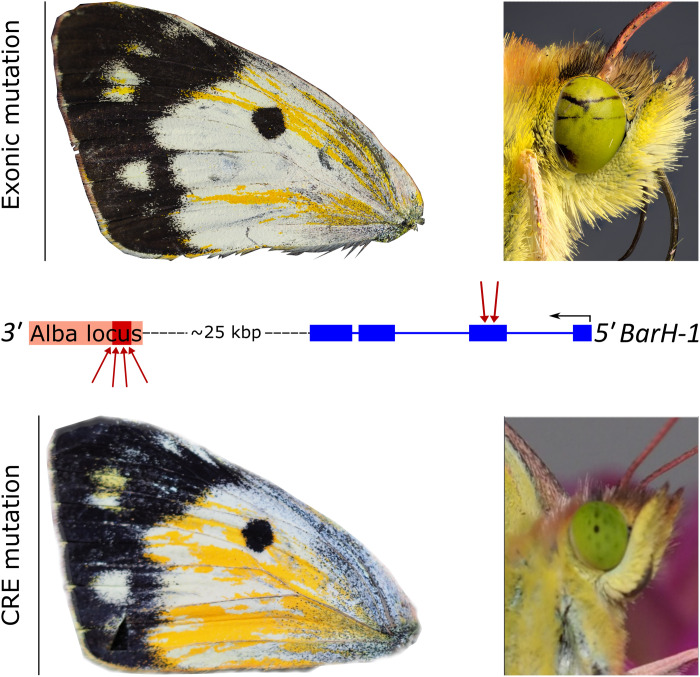
Comparison of CRISPR-Cas9 somatic mosaic knockouts at the *Alba* candidate locus. The top row illustrates the phenotypic effect of mosaic deletions in the second exon of *BarH1* (downward pointing red arrows onto second exon in blue), in a single representative Alba female wing and eye, made by Woronik *et al.* (*28*). The bottom row illustrates the result of mosaic deletions at the *Alba* locus made in this study, which is >25 kbp downstream of the *BarH1* gene. In the middle row, the target locations of the CRISPR-Cas9 gRNAs used in both studies to generate double-stranded breaks are depicted (red arrows) relative to the *BarH1* gene model (blue, exonic) and the 1200-bp *Alba* locus (pink, and the same window as in Fig. 4, with deletions indicated in red). Because deletions in the *Alba* locus cause similar changes in wing color to deletions in the *BarH1* coding region, while aberrations in the eye are only seen in the latter, we conclude that the *Alba* locus harbors a tissue-specific CRE necessary for the Alba wing color phenotype.

Our comparative analyses of the *Alba* candidate locus found that all Alba females share a conserved ~1200-bp insertion downstream of the TF *BarH1* (Figs. 3 and 4). In *C. crocea*, it is known that BarH1 is present in the developing wings of Alba, but not orange, individuals and that *BarH1* knockout results in a wing color change from white to orange (*28*). However, whether this *Alba*-specific insertion is a cis-regulatory element (CRE) that drives the observed difference in BarH1 expression that is associated with Alba females remains unknown. To test this hypothesis, we induced a somatic deletion mosaic of the conserved *Alba* candidate locus, using CRISPR-Cas9 gene editing in *C. crocea* (pink highlighted region in Figs. 3C and 4). Guide RNAs (gRNAs) were designed to target a region of the conserved *Alba* candidate locus that contained a large number of putative TF-binding sites, including for doublesex (fig. S21 and table S9). Along with Cas9, four gRNAs targeting different parts of the locus were injected individually and together as a cocktail to generate multiple cuts and remove 1 to 200 bp across its 1.2-kb length, including the putative *dsx*-binding site (fig. S16). While injections with a single gRNA did not produce any phenotypic changes (table S8), the cocktail containing all four gRNAs was successful. Two genetically Alba females (fig. S15) reached adulthood, both of which exhibited extensive wing phenotypes where scales recovered the orange pigmentation of non-Alba females (Fig. 6 and fig. S11). Successful mutagenesis was confirmed by PCR fragment size polymorphism relative to uninjected Alba females and amplicon sequencing (figs. S15 and S16). Thus, our knockout of the candidate CRE region produced wing phenotypes that are a phenocopy of the effects seen in previous mosaic knockouts that targeted the coding region (exon 2) of *BarH1* (*28*). However, while the previous knockouts in the coding region of *BarH1* interrupted the role of BarH1 in eye development in males and females of both morphs (*28*), none of our CRE knockouts in the putative cis-regulatory region produced detectable eye phenotypes (Fig. 9, table S8, and fig. S15). To verify this, eye tissue was genotyped to confirm the presence of CRE knock-out cells in individuals with normal eye phenotypes (fig. S16). Although our sample is small, this result (Fig. 9) is consistent with the expected lower pleiotropic impacts from targeting a modular CRE compared to the coding region of a gene (*45*). Additional study is needed to document the full extent of the pleiotropic effect differences between CRE versus coding knockout effects.

## DISCUSSION

We are able to reject the hypothesis of multiple independent origins of Alba for nearly the entire genus of *Colias*. While we cannot identify the maximum age of Alba at this time, our results indicate that it evolved at least 1.5 million years ago, before the separation of the North American and Eurasian clades (Fig. 1E). Since most *Colias* have at least one generation per year, this polymorphism has been maintained in at least as many generations. However, given the presence of Alba in South American taxa (*46*), Alba is likely to be much older (e.g., the age of *Colias*). Thus, the Alba ALHS is an ancient, trans-specific polymorphism. Whether other systems harboring polymorphisms across species radiations also have a simple genetic basis with a single, shared origin remains to be seen, but our finding suggests that these instances may not be rare (*5*).

Now that we know Alba is orthologous among diverse, divergent species of *Colias*, we wonder how this ALHS has been maintained within, and moved among, species so readily. In contrast to other well-documented systems where either introgression or balancing selection have been suggested to be the principal mechanisms maintaining allelic variation across diverse species within a genus [e.g., mimicry alleles in *Heliconius* and *Papilio* butterflies (*13*, *47*), respectively], we do not see evidence of such a clear distinction. Instead, our results suggest that the maintenance of Alba among species in *Colias* has been due to an interplay of introgression and balancing selection. However, rather than arguing that ALHS phenotypes might be experiencing different selection dynamics than mimicry phenotypes, we wish to draw attention to differences in the genomic architecture of these traits. In a recent review on the persistence of polymorphisms across species radiations, Jamie and Meier (*5*) summarize diverse empirical and theoretical studies, concluding that polymorphic traits likely have a simple genetic basis, which allows easy movement across species. We further suggest that the extent of negative pleiotropic interactions of such a locus also affects movement across species. A large-effect allele at a cis-regulatory locus will likely move among taxa much easier than alleles composed of divergent coding exons, since the latter will affect every instance a gene is expressed, while the former can have extremely narrow phenotypic effects. This could account for why mimicry and ALHS alleles in *Heliconius* and *Colias* appear to move across species with relative ease, while mimicry alleles in *Papilio* appear to rarely, if ever, introgress among taxa.

*BarH1* is a critical component of insect development and functions across diverse tissues from eyes to limbs (*48*–*50*), as well as being expressed in adult insects with potentially important regulatory roles (*51*, *52*). Successful co-option of *BarH1* for the Alba phenotype might have long ago been selected to reduce antagonistic outcomes, likely via narrow spatiotemporal regulation in its novel context (*53*). Consistent with the expectations, our findings suggest that *Alba* is a cis-regulatory locus that is functioning as a modular enhancer, inducing alternative strategies with minimal antagonistic pleiotropy (*54*). To what extent BarH1 affects the ALHS beyond its role in wing development is unknown, but our advances here in manipulating a large and potentially core component of an ALHS now allow detailed testing of whether the Alba ALHS arises via a simple trade-off of resources mediated by pigment formation, versus via additional roles of this regulatory region, in other time points and tissues, and potentially additional genes. Last, whether the modularity we observe for the *Alba* CRE is a general feature of ALHS alleles, or a requirement for ones maintained by introgression across diverse species, awaits further study of Alba and the development of functional genomic tools in other natural systems with ALHS.

## MATERIALS AND METHODS

### *C. eurytheme* genome

High–molecular weight DNA from six female pupae originating from Davis (CA, USA) and reared in the laboratory for several generations was sequenced on PacBio Sequel v1 at the University of Maryland-Baltimore Institute of Genomic Sciences. Assembly followed the Falcon/Falcon-Unzip/Arrow assembly pipeline (*55*) and led to a diploid genome length of 583 Mb, with N50 of 2.7 Mb. Haploidization was performed using Haplomerger2 (*56*), generating a haploid genome of 364.5 Mb with 123 scaffolds.

### *C. eurytheme* genome polishing, quality control, and annotation

Pilon v.1.22 (*57*) was used to polish the genome, using 150-bp paired-end (PE) reads (350-bp insert, Illumina HiseqX) aligned with NextGenMap v.0.5.2 (*58*). Genome quality before and after polishing was assessed using Busco v1.1b1 with OrthoDBs Lepidoptera v10, as well as N50 (*59*, *60*). Repetitive regions were softmasked using RED v:05/22/2015 (*61*). The genome was annotated using the Braker2 pipeline (*62*) with transcriptome data generated in a previous study (*63*) aligned with Hisat2 v2.2.1 (*64*) and protein data from OrthoDBs Arthropoda database (V10).

### Synteny comparative analysis

To assess our genome assembly and check for any large-scale structural changes compared to other sequenced lepidopteran genomes, we compared our *C. eurytheme* chromosome to one from the sister genus, *Zerene cesonia* (*65*). Whole-genome alignments were performed using nucmer v4.0 (*66*) followed by circos plotting using the R package circlize v.0.4.9 (*67*).

### Colias phylogenetic analysis

For each individual [21 species, one to three samples per species, one to two locations per species (see table S2)], whole-genome sequencing reads were generated via DNA extracted from thorax and/or abdomen via a salting-out method (*68*). DNA quality was evaluated using a 260/280 ratio (NanoDrop 8000 spectrophotometer; Thermo Fisher Scientific, Waltham, MA, USA). The library preparation and short-read PE sequencing (500-bp insert) for all individuals was performed at BGI China. Reads were filtered for adapters and trimmed at the 5′ and 3′ end based on a PHRED quality score > 20 using BBtools v38.08 (*69*). Reads were aligned to the *C. eurytheme* reference genome using NextGenMap v0.5.5 (*58*). Using these bam files after MAPQ > 20 filtering via Samtools v.1.9 (*70*), the longest exon per gene for each BUSCO gene, from each individual, was obtained from the CDS annotation for *C. eurytheme* via bam2fasta script from the package bambam v1.4 tool kit (Supplementary Materials) (*71*). *Z. cesonia* (*65*) was used as an outgroup, the dataset of which was generated by aligning Illumina sequencing reads from *Z. cesonia* (SRR11021459) to the *C. eurytheme* genome, as per the bam2fasta pipeline outlined above. Individual gene trees were then estimated using iQTree v.2.0.6 (*72*), which were then used to estimate a species tree via ASTRAL v.5.7.3 (*73*). Gene tree support for the species tree was assessed using Phyparts (https://bitbucket.org/blackrim/phyparts/src/master/). Species trees for each chromosome were generated using all genes trees of a given chromosome to generate an Astral species tree. SNAPP v.1.3.0 (*74*) analysis, implemented in BEAST2 v.2.6.3 (*75*) using SNPs randomly drawn from this gene set with a reduced taxonomic sampling, followed previous extensive analyses for optimal analysis settings (*33*), with dataset construction using snapp_prep.rb (https://github.com/mmatschiner/snapp_prep, accessed on 22 April 2021). Calibration for the timing of the split between *Zerene* and *Colias* used a secondary calibration of 10.9 million years ago (*32*), along with two monophyletic constraints set to increase run speed (South America taxa and non–South America taxa). See the Supplementary Materials for more details.

### Introgression analysis

Introgression between different species was estimated using D-statistics calculated from ABBA-BABA between all possible trio combinations using the Dsuite software package v0.3 (*76*). Using Dsuite, we also calculated an f-branch metric, a statistic related to the f-4 statistics, which allows summarization of the amount of shared introgressed material on a branch and infers past gene flow (*35*). Using the Alba reference genome, we aligned the reads of each species using NextGenMap and then called variants using Freebayes (*77*). The resulting vcf file was filtered (see the Supplementary Materials for details) using vcftools (*78*). Then, using the Dinvestigate tool part of the Dsuite tool kit, *d*_f_ and f_dM were calculated in nonoverlapping windows to look for signals of adaptive introgression along the chromosomes (*79*). RND was estimated using *D*_xy_ estimates calculated via Pixy v.1.2.4.beta1 (*80*) in nonoverlapping windows of 10 kb from an allsite-vcf generated using bcftools following recommendations in the Pixy manual. RND was calculated by dividing the *D*_XY_ of the target species pair with the average *D*_XY_ of each species with a shared outgroup species {*D*_XY_/[(*D*_XO_ + *D*_YO_)/2]}. In both RND analyses, *C. crocea* was used as an outgroup. The resulting dataset had all infinite values removed (caused by regions where *D*_XO_ or *D*_YO_ was 0). Additional details are provided in the Supplementary Materials.

### *C. eurytheme* × *C. philodice* 2b-RADseq genotyping and linkage map

We created a linkage map using genotypes generated by 2b-RADseq (*81*), of an F2 brood from a *C. eurytheme* × *C. philodice* hybrid cross resulting in genome-wide genotypes. These genotypes were used for linkage mapping following the basic LepMap3 protocol with some exceptions (explained in the Supplementary Materials). This resulted in 31 linkage groups, with one short unplaced scaffold. Since the Alba phenotype segregated among the female individuals in the cross (and they were reared to adults so they could be phenotyped), we were also able to identify the chromosome carrying the *Alba* locus (females lack recombination in Lepidoptera, and the female in the cross donated the *Alba* allele). To do this, the linkage map was output as a four-way cross, which was imported into R package r/qtl using custom code (contributed by K. Broman). We performed a genome scan with a single quantitaitve trait locus (QTL) binary model and ran a permutation test (*n* = 1000) to determine a 5% significance threshold.

### GWAS of Alba in *C. eurytheme*

Individuals used in the genome resequencing were from 15 Alba and 14 orange *C. eurytheme* females caught in 2012 near Tracy, California, and subsequently stored at −20°C in 95% ethanol. For DNA extraction through to read cleaning, see the Supplementary Materials. Cleaned reads were mapped to the *C. eurytheme* reference genome using NextGenMap v0.5.2, followed by duplicate marking, and then Freebayes v1.3.1-16-g85d7bfc for variant calling. The variants were filtered using VCFTOOLS v0.1.13 (*78*). Variants were associated with the Alba phenotype using PLINK v1.9 (*82*). Two separate sets of filters were used, one with stronger priors, where the nature of the inheritance pattern was taken into account, and one with weaker priors, where sites were filtered primarily by quality and depth; for more detailed information on the filters, please refer to the Supplementary Materials.

### Generation of an Alba-specific reference genomes

To characterize the sequence and structure of the *Alba* insertion, we generated an Alba reference genome. First, we generated a draft genome using a 10X Chromium library, sequenced on a NovaSeq S4, 2 × 150 bp PE reads, followed by assembly with Supernova v2.1.1 (performed by SciLifeLab). In addition to *C. eurytheme*, we also generated draft genomes for *C. nastes* (a species fixed for Alba), as well as *C. crocea* (used as a control to compare against the previous genome using 10X technology), using the same protocol.

### GWAS using the Alba reference genome

Using the new *C. eurytheme* Alba reference genome, we repeated the steps done in the initial GWAS, seeing if the alternative loci disappeared with new targets to map against.

### Characterizing the Alba insertion in *C. eurytheme*

We identified the scaffold containing *BarH1* by using tBLASTn in the *C. eurytheme* Alba Supernova assembly. We then aligned all the resequencing data from the GWAS to this contig. Read depth along the contig was analyzed visually in IGV, and differences between the orange and Alba morph were noted. Regions where no orange reads aligned, but Alba did, were extracted and blasted back against the *C. crocea* reference genome (*28*) to assess whether this was the previously identified *Alba* insertion region. To identify borders of the *Alba* insertion in *C. eurytheme*, we aligned the Alba contig against the orange *C. eurytheme* reference genome using BLASTn. This provided the boundaries of the *Alba *insertion region for *C. eurytheme*, which was then used to place this haplotype into the orange *C. eurytheme* reference genome assembly, creating what we refer to as the *C. eurytheme* Alba reference genome.

### Balancing selection and estimating the allele frequency of indels in *C. eurytheme*

Estimates of Fst, Dxy, Tajima’s D, and nucleotide diversity were made from the GWAS samples. First, an invariant site vcf was generated using Bcftools v.1.13-35-ge3ba077 (*83*). The subsequent vcf file was filtered to remove indels and sites with a quality score lower than 20. To calculate Tajima’s D, we used vcftools, in window sizes ranging from 10 to 50,000 bp, and differences between orange and Alba in Fst, Dxy, and nucleotide diversity were subsequently calculated using Pixy (*80*). Betascan2 was run only on scaffold 2, where the *Alba* insertion is located, using the recommended settings.

We called structural variants in all *C. eurytheme* samples used for the GWAS using the default pipeline in DELLY v.0.8.1 (*84*). Variants were first called in each sample individually, then merged, and genotyped jointly. The final output was first filtered using the germline filter provided by DELLY and second by including only sites in which no samples had a low-quality call (not enough read support to genotype).

### PCR-based validation of insertion

The presence and uniqueness of the insertion to Alba individuals were validated using PCR-based markers with primers designed to bind within the insertion region. Primers were designed using primer3 software (libprimer3 release 2.5.0). DNA from eight orange and eight Alba *C. eurytheme* females was used in the analysis, from the same 2012 field collection, and mtDNA cytochrome c was used as a positive control in each reaction. For more information about primer design and the reaction, see the Supplementary Materials.

### Identification of Alba insertion in *C. nastes*

The genome assembly was scanned for the presence of the *C. crocea* insertion sequence, as well as the sequence identified in the *C. eurytheme* Chromium assembly, and whether it was found in linkage with the *BarH1* gene using the same combination of tBLASTn and BLASTn as we used in *C. eurytheme*.

### Alignment and assessment of the Alba insertion across species

Resequencing data generated for the phylogeny, as well as from *C. philodice* from a *C. eurytheme–*hybrid population in Maryland, generated for (*85*) and *C. crocea* from (*28*), were aligned to the Alba reference using NextGenMap, filtered for MAPQ ≥ 20, having proper pairs, and for having coverage across the *Alba* candidate locus. We assessed whether the presence of read coverage segregated with female wing color. Regions that were unique to only white-colored species (putative Alba ALHS species) were considered to be conserved regions of the *Alba* insertion and likely causal for the phenotype. The likelihood of coverage segregating between the two color morphs to this degree was estimated using a window-based analysis (see the Supplementary Materials).

### Phylogenetic relationship of Alba insertion

We extracted the consensus sequence of reads aligned to the Alba candidate locus with the bam2fasta tool (part of the bambamv1.4 tool kit) and assessed their phylogenetic relationships using IQtree with the same settings as in the primary analysis (Supplementary Materials). Because of large amounts of repetitive content in the flanking regions, we limited the phylogenetic analysis of the *Alba* insertion to the *Alba* candidate locus.

### CRISPR-Cas9–targeted mutagenesis of the Alba insertion

PROMO v.3.0.2 (*86*, *87*) was used to scan the conserved *Alba* locus for potential TF-binding sites. The motifs were compared against version 8.3 of the TRANSFAC database (for output, see table S9). The output of this scan was analyzed in three ways: (i) binding sites of relevant candidate TFs were identified, such as *doublesex;* (ii) sites with a high density of potential TF-binding sites were recorded; and (iii) sites that were highly conserved between different species were preferentially selected. We designed four single-guide RNAs (sgRNAs) seeking to produce multiple cuts and produce a large >100-bp deletion (table S7).

Two of the sgRNAs were designed to specifically target the predicted *dsx* binding site, and two were located flanking *dsx* approximately 200 bp in either direction. In addition to *dsx*, the area in between the flanking sgRNAs includes the highest density of predicted TF-binding sites in the *Alba* locus (table S9 and fig. S21). gRNA design and injection were performed following the steps outlined by Woronik *et al.* (*28*) and injected as a cocktail together with Cas9 at a concentration of 500 ng/μl. *C. crocea* Alba females (*n* = 6) from Aiguamolls de l’Empordà, Spain, were captured, transported to Stockholm alive, and allowed to oviposit on *Vicia villosa*. Eggs were collected three times daily for injection, ensuring that they were not more than 4 hours old at the time of injection. Injected eggs (*n* = 200) were kept on glass slides inside sealed petri dishes, together with moist paper. Hatched larvae were transferred to fresh *V. villosa* and kept in feeding cups with no more than five larvae at 23°C until pupation. Once pupated, the pupae were transferred to a climate cabinet kept at 16°C until eclosion. Individuals with wing color phenotypes were assessed for CRISPR by sequencing PCR amplicons of the cut site using Nanopore sequencing. Each amplicon was sequenced using a ligation-based LSK109 library kit using the manufacturer’s recommended methods and sequenced until at least 100,000 reads were generated.

### Butterfly stocks

All animals were reared in accordance with Stockholm University institutional guidelines.

## Supplementary Materials

### This PDF file includes:

Supplementary Text Figs. S1 to S25 Table S1 Legends for tables S2 to S9 References

### Other Supplementary Material for this manuscript includes the following:

Tables S2 to S9

ST_covstat

## Notes

View/request a protocol for this paper from *Bio-protocol*.
