# Supplementary material for: Evidence for a single, ancient origin of a genus-wide alternative life history strategy
Source: Sci Adv. 2023 Mar 22;9(12):eabq3713. doi: 10.1126/sciadv.abq3713 (PMC10032607; doi:10.1126/sciadv.abq3713)
Supplement: Supplementary file 1 — Supplementary Text Figs. S1 to S25 Table S1 Legends for tables S2 to S9 References [file sciadv.abq3713_sm.pdf]

Science Advances
AAAS

Supplementary Materials for

Evidence for a single, ancient origin of a genus-wide alternative life history strategy

Kalle Tunström *et al.*

Corresponding author: Kalle Tunström, kalle.tunstrom@gmail.com;
Christopher W. Wheat, chris.wheat@zoologi.su.se

Sci. Adv. **9**, eabq3713 (2023)
DOI: 10.1126/sciadv.abq3713

The PDF file includes:

Supplementary Text
Figs. S1 to S25
Table S1
Legends for tables S2 to S9
References

Other Supplementary Material for this manuscript includes the following:

Tables S2 to S9
ST_covstat

Supplementary text

Bioinformatic scripts

Detailed scripts for bioinformatic analysis, if not specified in the relevant section, be found on zenodo (DOI: 10.5281/zenodo.7594986).

Summary of Colias eurytheme genome assembly

A chromosome-level genome for *C. eurytheme* was generated and used as a reference for aligning whole-genome sequence data from 21 species representing the global distribution of *Colias* and diverse Alba phenotypes. After haplomerging and polishing, the final haploid genome was 328 MB, with an N50 of 5.2MB across 108 scaffolds, high gene completeness, and low duplication (97.7% of expected single-copy genes were complete and unique, i.e., 5166 of 5286 BUSCO genes; Table S2). Using RAD sequencing data from a *Colias eurytheme* x *Colias philodice* F2 cross (*88*), we then generated and used a linkage map to assemble 31 chromosomes, which is the expected number for most *Colias* species (*89*). The *C. eurytheme* chromosomal structure was highly syntenic with the standard organization of Lepidoptera chromosomes (Fig. S3). Annotation of the resulting chromosome level assembly identified 18,077 transcripts from 16,842 genes.

Colias eurytheme DNA extraction and genome sequencing:

A *Colias eurytheme* stock originating from Davis (CA, USA) was maintained in a laboratory setting for several generations. High molecular weight DNA from six female pupae was isolated using the Qiagen Genomic-tip 100/G. The specimen yielding the most DNA (574.4 nanograms per microliter) was submitted for quality control, BluePippin extraction fragments > 15kb, PacBio SMRTBell Express library preparation, and PacBio Sequel v1 sequencing at the U. Maryland – Baltimore Institute of Genomic Sciences. Six PacBio Sequel v1 cells were sequenced and yielded a total of 50.46 Gb of sequence data with an average sub-read length of 10kb, *i.e.,* a coverage of 144x assuming a genome size of 350 Mb based on flowcytometry. Assembly of the data using the Falcon/Falcon-Unzip/Arrow assembly pipeline (*55*) was outsourced to DNAnexus (Mountainview, CA), which led to a diploid genome length of 583 Mb with an N50 of 2.7 Mb. Haploidization of the genome was performed using Haplomerger2, leading to a haplogenome of 364.4 Mb assembled into 123 scaffolds with a scaffold N50 = 4.82 Mb.

C. eurytheme x C. philodice 2b-RADseq genotyping and linkage map

To further improve the assembly and generate chromosome-wide super-scaffolds, a linkage map of the genome was generated using the 2b-RADseq (*81*) whole-genome genotypes of the F2 brood from a C. *eurytheme* x *C. philodice* hybrid cross (*88*). DNA was extracted from the thorax of frozen individuals using a bead-shaker and the Quick-DNA 96 Kit (Zymo Research, Irvine, CA, USA). 400 ng of DNA per individual was digested with the *BcgI* enzyme (New England Biolabs, Ipswich, MA, USA), and the 36 bp restriction fragments were purified using the ZR-96 Oligo Clean & Concentrator kit (Zymo Research). Barcoded adapters corresponding to a 16 reduced tag representation were added (*81*) before ligation with the NEBNext Multiplex Primers Set 1-4 (New England Biolabs). The pooled library was enriched

for a 155-175 bp (target inserts of 166bp) using a BluePippin instrument (Sage Science, Beverly, MA, USA) and sequenced using an Illumina HiSeq4000 SR50 run.

We investigated whether the haplogenome still contained some haplotype scaffolds/contigs. To achieve this, the two first steps (hm.batchA1.initiation_and_all_lastz + hm.batchA2.chainNet_and_netToMaf) of Haplomerger2 (*56*) were run on the haplogenome to create the alignment chain (all.chain.gz). The alignments of the remaining 123 scaffolds revealed that there were some haplotypes left. We manually classified all scaffolds into full, partial, or unique scaffolds; there were 16 full and 11 partial haplotypes. Using a custom script, the remaining haplotypes and partial haplotypes were removed from the assembly to construct the final scaffold assembly with 108 scaffolds (one scaffold was cut to two, as it was likely a chimera).

The genotype data for the linkage map for the *C. eurytheme* genome was obtained by Lep-MAP3 (LM3)(*90*) pipeline. First, the individual fastq-files were mapped to the scaffold assembly using bwa mem (*91*) and using LM3 pipeline (pileupParser.awk, pileup2posterior.awk) and SAMtools mpileup (*70*), we obtained the input genotype likelihoods.

Linkage mapping followed the basic LM3 pipeline as follows (non-default parameters inside parenthesis):

1. ParentCall2(ZLimit=2, removeNonInformative=1)
2. Filtering2 (dataTolerance=0.0001)
3. SeparateChromosomes2(lodLimit=14.5 maleTheta=0.5 femaleTheta=0.0001 distortionLod=1 sizeLimit=4)
4. 2 x JoinSingles2All (lodLimit=10 lodDifference=2 maleTheta=0.05 femaleTheta=0.0001 distortionLod=1)
5. OrderMarkers2 (chromosome=1..31 recombination2=0 informativeMask=13 useMorgan=1).

The above pipeline yielded 31 linkage groups, but two linkage groups were joined after inspection revealed that the markers occurred in the same scaffolds (27+30 and 29+25). Moreover, two linkage groups had very long maps (>100cM) and these groups were split, group 1 with SeparateChromosomes2 (map=map14.5.txt maleTheta=0.5 femaleTheta=0.0001 distortionLod=1 lg=1 renameLGs=0 lodLimit=16) and group 10 based on sex/autosome markers (indicated by * in the output of ParentCall2).

After these splits and joins, the JoinSingles2All and OrderMarkers2 were rerun to obtain final (*de novo*) linkage maps with 31 linkage groups. The splits and joins were necessary due to the complex family structure (multiple families) and low marker density.

With the help of the linkage map, the scaffolds (all except Sc0000116) were manually put together into these 31 linkage groups. Linkage groups were named based on chromosome numbers in *Melitea cinxia* (*92*). The linkage map was re-evaluated in this physical order with OrderMarkers2 (recombination2=0 chromosome=1..31 evaluateOrder=phys_order improveOrder=0 hyperPhaser=1 phasingIterations=3), put into grandparental phase (phasematch.awk) and this map was used for QTL mapping.

C. eurytheme genome polishing, quality control, and annotation

Polishing of the genome was performed using Pilon v1.2.2 (*57*), using data from a single orange female, with an Illumina TruSeq Nano library prep and sequenced (150 bp PE reads with a 350bp insert size, Illumina HiSeqX) to provide ~30X genome coverage, aligned using NextGenMap v0.5.2. The assembly quality was assessed using basic length metrics and BUSCO v1.1b1 before and after polishing to evaluate the difference, using the insecta_odb10 dataset. The genome was softmasked for repetitive regions using RED v.05/22/2015 (*61*).

Genome annotation was generated using BRAKER2 (v2.1) trained on data from *C. eurytheme* transcriptome and proteins. The transcriptome was assembled using RNA-seq data from several developmental life stages that was generated in a previous study (*63*). We used a reference protein dataset from the Arthropoda section of OrthoDB (v10). Transcriptome reads were aligned using HISAT2 v.2.1.0 (*64*), against the unmasked genome, and the alignment was then filtered, sorted, and indexed using SAMTOOLS v.1.7. Braker2 was run using the ETP mode and set to take softmasking into account. Annotation of the resulting chromosome level resulted in 18077 transcripts from 16842 genes.

Synteny comparative analysis

To assess our genome assembly and check for any large-scale structural changes compared to other sequenced lepidopteran genomes, we compared our *C. eurytheme* chromonome to one from the sister genus, *Zerene cesonia* (*65*). Whole-genome alignments were performed using nucmer followed by circos plotting using the R package circlize v.0.4.9 (https://academic.oup.com/bioinformatics/article/30/19/2811/2422259).

Balancing selection analyses

In order to estimate Fst, Dxy, nucleotide diversity, and Tajima's D from the *C. eurytheme* samples, we first generated an invariant-sites vcf to avoid having no- or low-coverage sites influence the statistics. We used bcftools mpileup and bcftools call variants (v. 1.13-35-ge3ba077)(*83*). We used the –TajimaD option in vcftools to calculate Tajima's in window sizes 10, 50, 100, 500, 5000, and 50000bp. To estimate differences in Fst, Dxy, and Nucleotide diversity between orange and Alba, we used Pixy (v.1.2.4.beta1)(*80*).

Beta statistics were calculated using Betascan2 (*43*), the previous VCF file, and default settings used for filtering the VCF file. We only analyzed scaffold 2, focusing the analysis to the region surrounding the Alba locus. Since scaffold 2 was 11 Mbp in length, with Alba located at 6.5Mb, this provided sufficient flanking region for a representative global analysis.

We called structural variants in all *C. eurytheme* samples used for the GWAS using the default pipeline in DELLY (*84*). Variants were first called in each sample individually using Delly call. Variants from each sample were then merged and genotyped jointly using delly merge, delly call and bcftools merge. The final output was first filtered using the germline filter provided by DELLY, and secondly by including only sites in which no samples had a low-qual call (not enough read support to genotype).

Phylogenetic analyses

The longest exon per gene dataset was generated by running BUSCO as above, but upon the protein dataset from our annotation of the *C. eurytheme* genome, with the protein dataset generated using our GFF annotation and the genome as inputs for the gffread script

from cufflinks v.2.2.1 (*93*). Of the total lepidopteran BUSCO genes searched (n= 5286), 4476 were complete and single copy in our protein dataset for *C. eurytheme*. Among the BUSCO outputs is a table where for each annotated protein identified, its BUSCO status is indicated (e.g., as single, complete, and duplicated, etc.). Using this table, the exons of the complete and single-copy proteins in our annotation were extracted and converted to a bed file. Then the length of each exon was calculated, allowing for the longest exon per protein ID to be selected using custom scripts, and the resulting bed file of these longest exons was then used as input for the bam2fasta script from the package bambam v1.4 tool-kit (*71*), along with all the bam alignment files and a minimum depth requirement of 5 reads per base pair. The resulting set of exons had a wide range of sizes (min,max exon lengths: 94,11667 bp). The resulting set of fasta-files (busco_exons) was then used to generate gene trees using iQtree, with each gene tree using extended model selection, a random starting tree, 1000 ultrafast bootstraps and optimization, and *Z. cesonia* set as an outgroup (-m MFP -t RANDOM -bb 1000 -alrt 1000 -bnni -o Z_cesonia). A total of 4244 gene trees were generated. These were then used as input for species tree estimates by Astral using default settings. Gene trees were also grouped by chromosome using the genome annotation, which allowed for species tree for each chromosome to be estimated.

We also generated a filtered busco_exon dataset, using AMAS V.1.0 (*94*) to remove *Z. cesonia* from all fasta-files, and then generate a summary table of all files, which was then parsed to produce a set of files filtered to remove those with missing content > 1 %, < 5 % variable sites), and length < 300 bp and > 2000 bp. With this filtered set of fasta file IDs (n=1400), these iQtree gene trees were then selected for species tree estimation to assess whether dataset quality affected species tree topology. The resulting species tree from these filtered fasta files was identical to the full busco_exon analysis. Additional analyses using complete CDS for ~9000 genes, or their second exon, produced essentially identical species tree results (Fig. S4).

Gene tree concordance with the species tree was assessed using Phyplots (*95*) to calculate the number of gene trees concordant and discordant with the species tree topology per node. Phyplots output was further parsed to distinguish among gene trees discordant with the species tree, into those supporting a main alternative tree vs. many alternative trees and those gene trees having less than 50% bootstrap support at the node in question. We used pieplots to represent these proportions
(
https://github.com/mossmatters/MJPythonNotebooks/blob/master/PhyParts_PieCharts.ipynb, accessed on 28/04/2021). Importantly, these pieplots results were concordant with estimated gene concordance factors via iQtree (Fig. S4).

We note here that the placement of the *C. philodice* Alba specimen from British Columbia (Canada) is grouping with *C. canadensis* instead of *C. philodice* from Connecticut (USA). Whether this represents a hybrid individual, paraphyly of *C. philodice*, the need to reassess the status of *C. canadensis*, or misidentification of the collected specimen from Canada, will be addressed in subsequent work.

SNAPP analyses (*74*), which are run as an add-on to the BEAST2 software program, were used to generate multi-species-coalescent analyses using SNPs from the busco_exon dataset from above. SNAPP estimates the species tree probability by integrating over all the possible gene trees observed among the SNPs. Despite dramatically decreasing parameter space for

analyses, this approach remains computationally demanding. Recent work has investigated the number of SNPs for optimal inference while minimizing computation demands and determined accurate SNAPP settings for time calibration, which uses a strict-clock model with fossil calibrations and the linking of all population sizes during analysis (*33*). Thus, in order to stay within a multispecies-coalescent framework for divergence time and phylogenetic relationship estimation, we followed these recommendations (*33*) and down-sampled our taxa to remove redundancy among closely related species while retaining regional diversity. For species where Alba was polymorphic and we sequenced both morphs, we only included the colored morph. This took our entire dataset of 29 samples down to 21. Using AMAS, exon fasta files were subsampled to these 21 species, concatenated, and then converted to phylip file format. A ruby script was used to generate an input XML file for SNAPP via snapp_prep.rb (https://github.com/mmatschiner/snapp_prep, accessed on 22/04/2021), which takes a phylip formatted sequence dataset, allows for specifications of run iterations, inter-SNP intervals, total SNPs, a starting tree, and various constraints to be incorporated. Constraints included a temporal calibration for the timing of the split between *Zerene* and *Colias* using a secondary calibration of 10.9 million years ago with sigma=1, along with two monophyletic constraints well supported by previous Astral analyses (one for South American taxa, one for the remaining *Colias* species). The SNP dataset was constructed drawing random SNPs from among the concatenated BUSCO exons with at least 300 bp between each SNP, monomorphic sites removed, as were non-bi-allelic sites, resulting in 131 bi-allelic sites.

In order to generate a starting tree, the exons in the busco_exon set were concatenated this full dataset run using iQtree (extended model selection, a random starting tree, 1000 ultrafast bootstraps and optimization, and *Z. cesonia* set as an outgroup (-m MFP -t RANDOM -bb 1000 -alrt 1000 -bnni -o Z_cesonia)). This concatenated exon tree shows nearly the identical topology to that of the ASTRAL gene tree (Fig. S19). From this, an ultrametric tree with branch lengths, and a cladogram tree without branch lengths, were used as the two starting trees for SNAPP runs. For each starting tree, four independent runs were initiated, each with 4 million iterations. All 8 resulting runs were assessed for convergence, which was determined by effective sample sizes (ESS) > 200 and convergent likelihood and posterior distributions, which was assessed using Tracer in the BEAST2 package. Tree files were combined using Treeannotator in the BEAST2 package, with posterior estimates using median tree credibility after 10% of data was discarded as burn-in. The resulting phylogenetic relationships and divergence estimates for *Colias* and non-South American *Colias* crown groups were nearly identical in their results, which were visualized using Figtree v.1.4.4 (*96*).

Introgression analysis

In order to assess the level of gene flow and introgression among *Colias* species, we calculated the D-statistic between all possible trios of species for which we had sequence data. Using the software Dsuite (*76*) we were able to analyze this jointly and infer between which, past and present taxa, have likely had introgression. By calculating a f-branch statistic and analyzing it together with the phylogeny, we can infer between which taxa and which nodes in the phylogeny gene flow is most likely.

For this analysis, we used the sequence data for different *Colias* species mentioned previously and aligned them to the manually curated *C. eurytheme* Alba-reference genome

using NGM (same settings as previously). No filtering was done before using Freebayes to call variants. The resulting vcf-file was filtered for strand-bias, a quality score of > 30, 90% of species sharing the site, minimum sample depth of 5, and minor allele frequency of 5%. In the analysis, only biallelic SNPs are included. D statistic between all our trios was calculated with the phylogeny as a reference tree to guide the analysis using the Dtrios tool in the Dsuite-toolkit (v.0.4). Dtrios compares all possible trios of species and calculates a genome-wide minimum D and a significance value of each trial, and it will also calculate F4 statistics for each trio that we used to calculate the f-branch statistic we used to infer signals of past introgression. To test if the difference in observed levels of introgression we observed between regions was driven by an incorrect placement of *C. phicomone* or *C. palaeno*, the two species whose placement differed between the SNAPP tree and the Astral tree, we removed these taxa from the analysis, however, while we saw a decrease in the strength of introgression by doing this, the general patterns persisted (Fig. S24 & S25).

GWAS of Alba in C. eurytheme

DNA was extracted using KingFisher Cell and Tissue DNA Kit (ThermoFisher Scientific, Waltham, MA, USA, N11997) and the robotic Kingfisher Duo Prime purification system (ThermoFisher Scientific, Waltham, MA, USA, N11997). DNA purity was assessed using 260/280 ratio (Nanodrop 8000 spectrophotometer; Thermo Scientific, Waltham, MA, USA), and concentration was quantified on a Qubit 2.0 Fluorometer (dsDNA BR; Invitrogen, Carlsbad, CA, USA). DNA was sent to the Science for Life Laboratory (Stockholm, Sweden) for library preparation and sequencing (Rubicon, 150 bp PE reads with 350bp insert size, Illumina HiSeqX). Sequencing libraries were sequenced twice to a predicted depth of 10x each time. Raw reads were clone filtered, had adaptors trimmed, and low-quality bases (PHRED 20) removed using the BBduk tool from the BBmap software package v34.86 (*69*). Cleaned reads were mapped to the *C. eurytheme* orange reference genome using NextGenMap v0.5.2. samtools was used to filter out unmapped reads, sort, duplicate marking of reads, and indexing. PICARD-tools v1.139 was used to add Readgroups and then again to merge the separate sequencing runs of each sample before a final round of duplicate marking using samtools. Variants were called by Freebayes v1.3.1-16-g85d7bfc (*77*), and the resulting VCF-file was filtered using a mix of VCFTOOLS v0.1.13 (*78*) and custom awk scripts. The final GWAS was run using PLINK v1.9 (*82*) to identify associated loci. We filtered the GWAS based on two separate levels of stringency. First, we filtered the VCF file to remove low quality SNPs or sites that did not match our depth or frequency criteria (minimum depth 3, minQ 20, max-missing 0.95, and minor allele cutoff of 0.05). Second, we added an additional filter to this set and added the prior criteria to include only sites unique to the Alba females. We also filtered the output using the information gained from the QTL analysis and only kept SNPs on the scaffolds that made up Chromosome 3.

The same filtering approach was also applied to the GWAS done using the Alba reference genome.

Generation of draft genomes of Alba individuals of C. eurytheme, C. nastes, and C. crocea

DNA from wild-caught females was extracted from the thorax of samples stored frozen in 95% ethanol using the same protocol as in the resequencing done for the GWAS. Prior to library preparation, the molecular weight of the DNA was estimated using gel electrophoresis (0.5% agarose LE). DNA was extracted from 2 individuals of each species, and the ones with the highest quality DNA (as determined by gel electrophoresis, the

260/280 ratio measured via a spectrophotometer (Nanodrop 8000; Thermo Scientific, Waltham, MA, USA), and DNA concentration quantified on a Qubit 2.0 Fluorometer (dsDNA BR; Invitrogen, Carlsbad, CA, USA)), were selected for library prep. Sequencing and assembly via Supernova v2.1.1 were performed at SciLifeLab (Stockholm, Sweden).

PCR-based validation of insertion

The presence and uniqueness of the insertion to Alba individuals was further validated using PCR-based markers. The primers were designed to bind within the insertion region, which was unique to Alba. Optimal primer binding sites were identified using the primer3 software v2.5.0 (*97*). PCR reactions were run on DNA extracted from 8 orange and 8 Alba females that had not yet been sequenced or used in the GWAS. Positive controls were run using previously validated primers binding to mitochondrial cytochrome oxidase I gene (*98*). The reactions were run using Invitrogen Platinum Taq in a Veriti 96-well thermocycler (Applied Biosystems, Foster City, CA, USA) using the recommended settings for the polymerase (72C x 2min followed 35 cycles of 94C x 30sec + 54C x 30sec + 72C x 15 sec followed by 72C x 5min). The PCR product was visualized by agarose gel electrophoresis in a 1% gel (Fig. S9).

Generation of the C. eurytheme Alba reference genome

We identified the scaffold containing *BarH1* in the supernova assembly of an Alba *C. eurytheme* using tBLASTn. We then aligned all the resequencing data from the GWAS to this contig alone and visually evaluated the contig in IGV. Regions where no orange reads aligned, but Alba did, were extracted and blasted back against the *C. crocea* reference genome (*28*) to assess whether this was the previously identified Alba insertion region. The one contig that both contained the *BarH1* gene and only had reads from Alba individuals mapping, was identified as orthologous to the previously identified *C. crocea Alba* insertion using BlastN. To identify the orthologous region of this contig in the orange *C. eurytheme* reference, we used BlastN. Once the regions in common between the Alba insertion contig and the orange reference were established, we manually inserted the entire Alba insertion contig into the orange reference genome. This resulted in what we refer to as the Alba reference *C. eurytheme* genome.

We decided to generate the additional *C. crocea* assembly to make sure that we would be able to assemble and subsequently detect the Alba insertion sequence. We used the same method here as we described in the main paper for the detection of the *Alba* allele in the *C. nastes* and *C. eurytheme* assemblies, where we blasted the previously known *Alba* allele detected in the *C. crocea* assembly (*28*), and if this insertion was co-located with the *BarH1* gene. We also assessed sequence similarity and synteny using Blastn, which we visualized using Kablammo (Fig. S10 & S11).

Alignment and assessment of the Alba insertion across species

The sequence data generated for the phylogeny was aligned to the Alba reference-genome using NextGenMap, filtered for mapQ 20, and being in proper pairs. Quantification of the proportion of mapped reads, and confirmation of sex in cases where it was uncertain, used a read coverage analysis via goleft indexcov (*99*) (see ST_CovStats). This confirmed that seven samples in our analysis were indeed male (*Colias erate poligraphus*, *Colias tamerlana mongola*, *Colias phicomone, Colias tyche, Colias behrii* (B033), *Colias tamerlana* (B037), and *Colias wiscotti*) based on the increased coverage on the scaffolds that make up the sex-chromosome (9, 19 & 52). It also emphasizes the divergence between the South American

species *C. lesbia* and *C. euxanthe* and the other species as they consistently showed highly reduced coverage on the terminal ends of the shorter chromosomes in the assembly (ST_CovStats).

Coverage across the insertion region was then visually inspected in IGV. We primarily assessed the regions where we had observed a difference in coverage between orange and Alba *C. eurytheme* individuals and whether this extended to other species where we had sampled either orange or Alba individuals. The sequence that was unique to only white-colored species (putative Alba species) was the conserved *Alba* region and likely causal for the phenotype across *Colias*.

To establish a null expectation for how often a region of the same size as the conserved *Alba* region would segregate in this manner between white and colored (e. g orange, yellow. red) species, we performed a read depth analysis of all the sequenced *Colias* species. Reads were aligned using NextGenMap and filtered for proper pairs. We then used goleft v.0.2.1 (https://github.com/brentp/goleft) to evaluate the average read depth of 600 bp windows across all scaffolds. We selected 600 bps rather than the full 1200 bp of the candidate *Alba* locus to ensure that at least one window ended up inside the insertion region; this also made the analysis more sensitive compared to using a larger window size. The read depth coverage in the windows was then classified in a binary fashion: having read coverage (1) or not (0), with the latter classification assigned if the window had less than 25% of the read depth compared to the scaffold average. Thus, for each window, each species had a value of 0 or 1. Then, using these values of either having reads covering the window or not, we calculated the mean value per window for the white and colored groups of species. Finally, we subtracted the white value per window from the colored value per window. For the *Alba* identified region in white-colored butterflies, this value was 1, which was then compared to the rest of the windows across the genome, which served as a genome-scale control.

Phylogenetic analysis of the Alba insertion.

We extracted the consensus sequence of the region from all 43 Alba samples in our analysis (14 *C. eurytheme,* ten *C. crocea*, seven *C. philodice* (Maryland), one *C. philodice* (from British Columbia), and one from each remaining Alba species in the phylogeny) using the bam2fasta 1.4 tool-kit. We kept all sites with at least a read depth of one and selected the most common allele at polymorphic sites. We then ran IQtree to generate a phylogenetic tree of the sequences. IQtree was run with model finder plus enabled to allow it to find the most parsimonious model.

CRISPR/Cas9 targeted mutagenesis of the Alba insertion.

PROMO v 3.0.2 (*86*, *87*) was used to predict transcription factor binding sites within the *Alba* candidate locus. Putative binding sites for proteins that have previously been shown to interact with *BarH1* or are known to be involved in sexually dimorphic phenotypes were identified. Of the candidates, a putative binding site of doublesex was particularly intriguing due to its known role in the development of a variety of sexually dimorphic phenotypes (*100*), including butterfly wing color (*101*, *102*). Thus, 4gRNAs that targeted the putative doublesex binding site were designed. Two gRNAs were designed to cut within the putative doublesex binding site, while the other two targeted upstream and downstream from the site (Fig. S21). Four types of cocktails of gRNA/Cas9 mixture were injected:

1. gRNA_P (within the doublesex binding site)

2. gRNA_P and gRNA_U (3bp upstream of the doublesex binding site)
3. gRNA_2 (~250bp upstream of gRNA_P) and gRNA_5 (~150 bp downstream of gRNA_P).
4. All four together.

Unfortunately, due to poor egg-laying of the Alba females, we could not inject an equal number of eggs with each combination, instead primarily injecting eggs with either gRNA_P alone or gRNA_U and gRNA_P. The only combination we saw any phenotype from was when we injected all four together. Unfortunately, we were only able to inject 40 eggs with this combination before the females stopped laying eggs. The primary cause of death among our injections was accidental, due to larvae sticking to the double-sided tape that we used to attach the eggs to the glass slide during the injection. Out of 200 injected eggs, we could only transfer 39 larvae to a fresh hostplant (5 of the 40 eggs we injected with all four gRNAs: 2 males, 1 orange and 2 Alba females); the remaining died from the trauma induced as we attempted to release them from the glue.

Validation of CRISPR/Cas9 targeted mutagenesis.

Using Primer3 (*97*) we designed PCR primer pairs that bind upstream and downstream of gRNA_2 and gRNA_5. Due to a large amount of repetitive DNA in the region, we were forced to have the forward primer bind to a non-unique location, leading to a risk of having some off-target binding sites. The reverse primer was unique, and there were no alternative bind-sites for the forward primer within 200Kb of the intended location. DNA was extracted using the Monarch HMW DNA extraction kit for Tissue (Cat# T3060S) using the front half of the thorax as well as from the eyes of both individuals with visible KO-phenotype. The PCR-reactions were run using Invitrogen Platinum Taq in a Veriti 96-well thermocycler (Applied Biosystems, Foster City, CA, USA) using the recommended settings for the polymerase (72C x 2min followed 35 cycles of 94C x 30sec + 54C x 30sec + 72C x 15 sec followed by 72C x 5min). The PCR products of the thorax samples were visualized on an agarose gel electrophoresis in a 1% gel (Fig. S15). We also used the primer pair developed by (*28*) to validate the Alba status of the sample, and as a positive control for the reaction as the two primer pairs do not bind near each other. We additionally sequenced the PCR products from the eyes and thorax of the two KO-individuals using Nanopore Minion. The PCR-product of each sample was individually cleaned using a 1.8x Ampure XP cleanup (Beckman Coulter, Brea, CA, USA) and prepared for sequencing according to the Nanopore LSK109 ligation library protocol. Each library was sequenced until approximately 100K reads had been generated. The reads were basecalled with guppy v6.0.1 using the super accuracy configuration and filtered for reads with a Q-score of >10. The resulting fastq reads were mapped against the *C. crocea* reference genome (*28*) and the location and size of deletions were visually identified and correlated to the target sites of the individual gRNAs used (Fig. S16).

Supplementary tables

Table S1. Quality metrics of the assembly

Size	
Contigs Generated:	108
Maximum Contig Length (Mb):	13
Minimum Contig Length:	38,228
Average Contig Length (Mb):	3.0 ± 2.9
Total Contigs Length (Mb):	327
N50 value MB:	5.2
Completeness	
BUSCO summary	C:98.5% [S:97.7%, D:0.8%], F:0.2%, M:1.3%,n:5286
Complete BUSCOs	5209
Complete and single-copy BUSCOs	5166
Complete and duplicated BUSCOs	43
Fragmented BUSCOs	12
Missing BUSCOs	65
Total BUSCO groups searched	5286
Annotation	
Transcripts	16842

Table S2. Metadata table of sequenced individuals, including capture site sample name.

Table S3. Top hits of GWAS after light filtering of SNPs, using the orange reference.

Table S4. Top hits using informed priors, using orange reference

Table S5. Top hits using QTL informed priors, orange reference

Table S6. Top hits using Alba reference genome and light filters. Note that the hits not on the *Alba* locus are on the sex chromosome.

Table S7. gRNA sequence

Table S8. Injection statistics and survival.

Table S9. PCR primers.

Supplementary figures

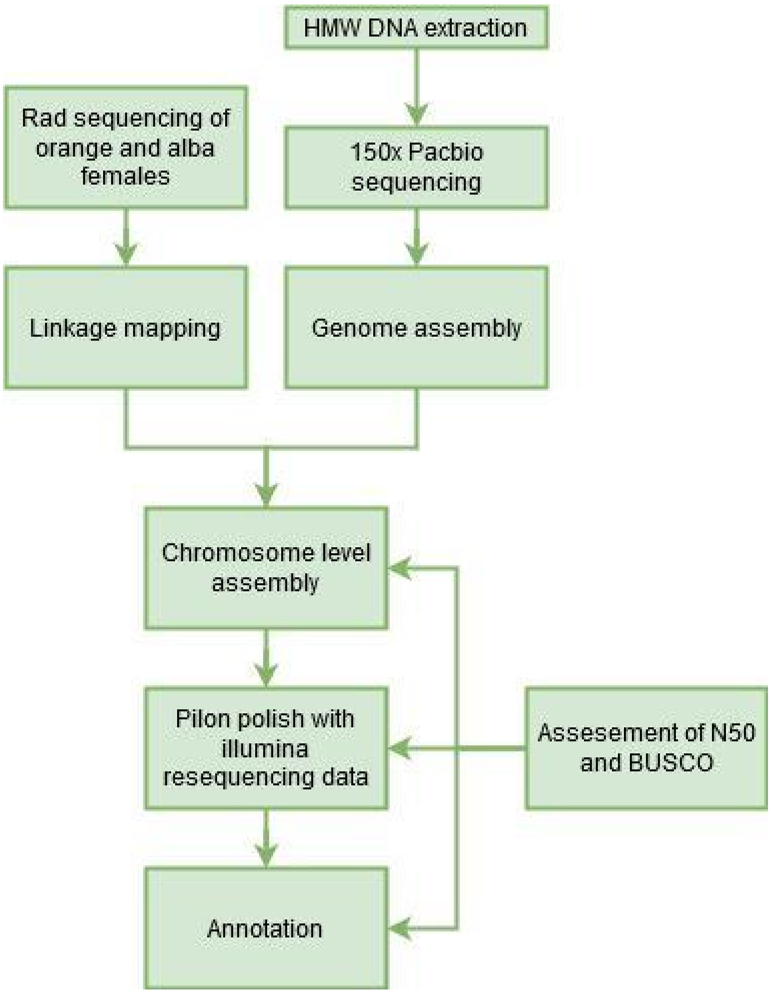

Fig. S1 Flowchart of the assembly pipeline.
Flowchart illustrating the genome assembly, annotation, and evaluation process for the *C. eurytheme* genome construction. Generation of raw contigs was done using 150x PacBio sequence data coming from a single orange female pupa. Linkage mapping of the contig data was done using Rad sequencing from multiple individuals of *C. eurytheme* X *C. philodice* hybrid crosses was used to create a linkage map of the contigs. The draft assembly was then polished using Pilon to reduce indel errors introduced by the PacBio sequencing,

and finally annotated using BRAKER2. Each step of the assembly process was analyzed using N50 and BUSCO.

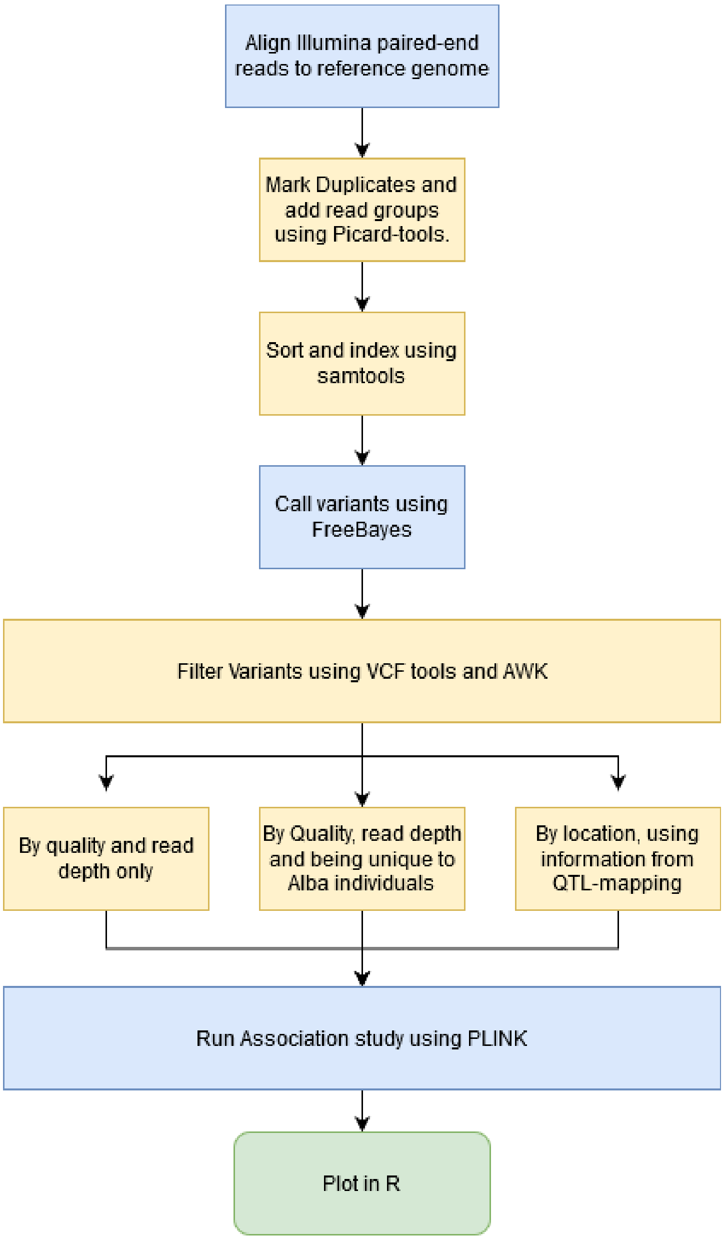

Fig. S2 Flowchart of variant calling pipeline for *C. eurytheme.*
Flowchart describing the steps involved in the Genome-Wide Association Study (GWAS). Blue boxes represent the generation of data, Yellow boxed filtering of data, and green boxes visualization. In total, we ran three levels of filtering of the raw VCF-file with different levels of priors.

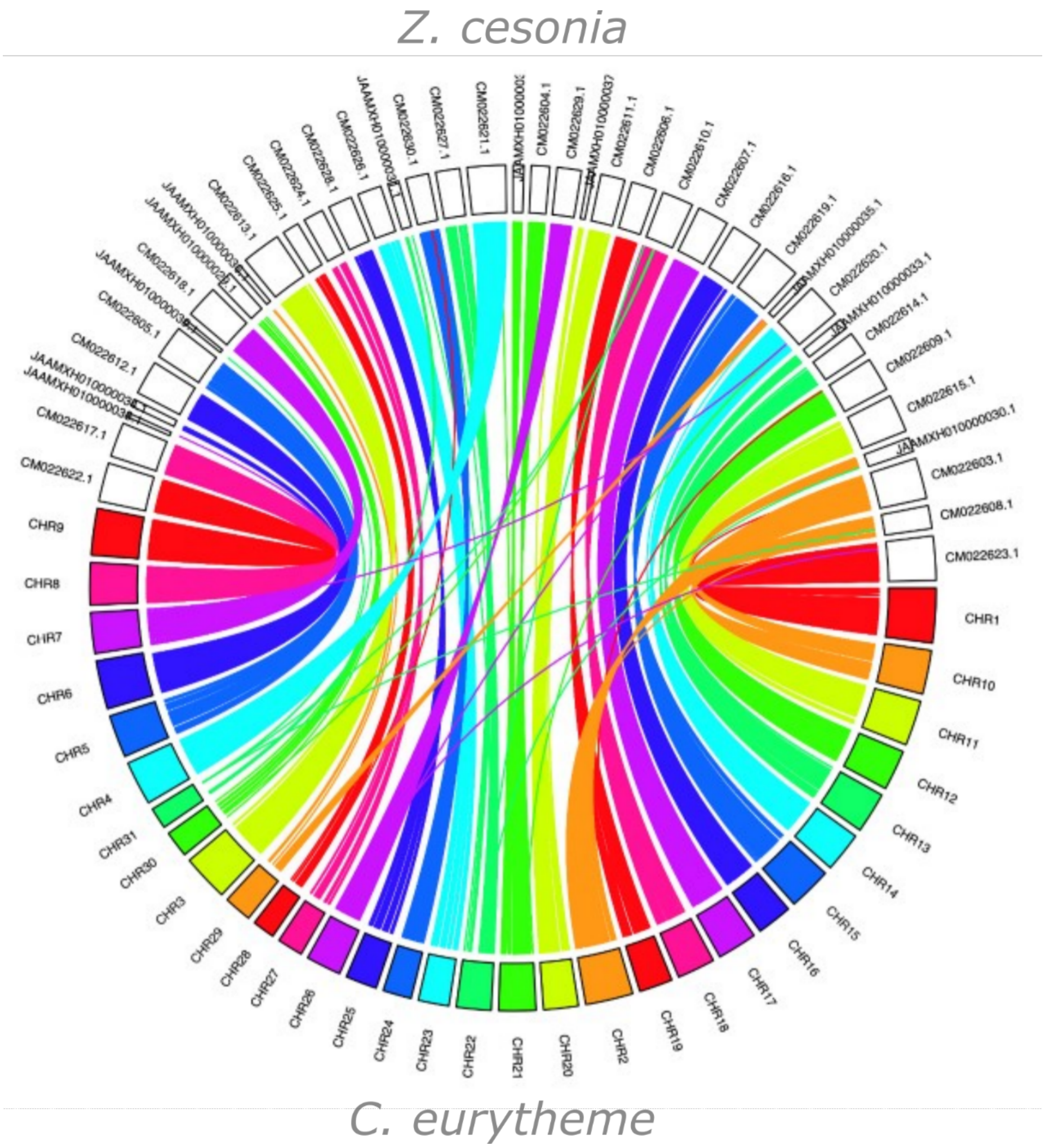

Fig. S3. Synteny plot of *C. eurytheme* assembly

Synteny plot illustrating the conserved chromosomal structural comparison between the Colias eurytheme genome and *Zerene cesonia*. The 31 linage groups identified in *C. eurytheme* are colored by chromosome, while the scaffolds of *Z. cesonia* are uncolored. Each line represents an inferred orthologous region. Only scaffolds > 1 Mb of *Z. cesonia* were included, with nucleotide alignment identity > 88 %. Note how several *Z. cesonia* scaffolds are brought together within *C. eurytheme* chromosomes (e.g., Chr 6 and Chr 29). Given the high synteny between *Z. cesonia* and *Heliconius erato* (*65*), and *Heliconius* to other butterflies and moths (*92*), we infer that Colias chromosomal structure adheres to the standard Lepidoptera chromosome structure (*103*).

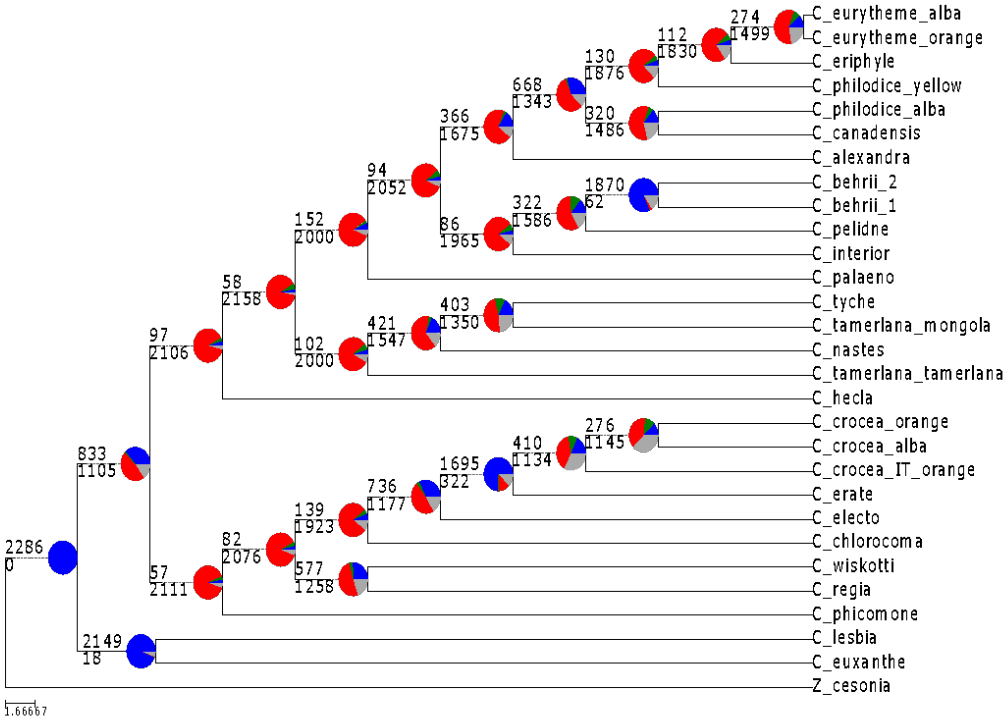

Fig. S4. Pie charts of gene tree concordance, conflict, and lack of signal compared to the Astral species tree. While traditional node support values, whether bootstraps or posterior probability, generally overstate gene tree support, here we present direct quantification per node of gene tree topology with the species tree topology. The number of gene trees concordant (top number) and in conflict (bottom number) is shown at each node. Pie charts at each node give a further breakdown of gene tree proportions by those concordant with species tree (blue), those that support a common alternative topology (green), those that support the remaining low-frequency alternatives (red), and those lacking robust information as they have less than 50% bootstrap support (gray). Here, the South American taxa are very well supported, as are the clades containing: *C. crocea*, *C. erate,* and *C. electo; C. tyche, C. tamerlana mongola, C. nastes*; *C. pelidne,* and *C. behrii*; *C. alexandra, C. canadensis, C. philodice, C. eurytheme,* and *C. eriphyle*. Note how the vast majority of nodes are red, indicating extensive gene tree conflict rather than a lack of phylogenetic signal (gray).

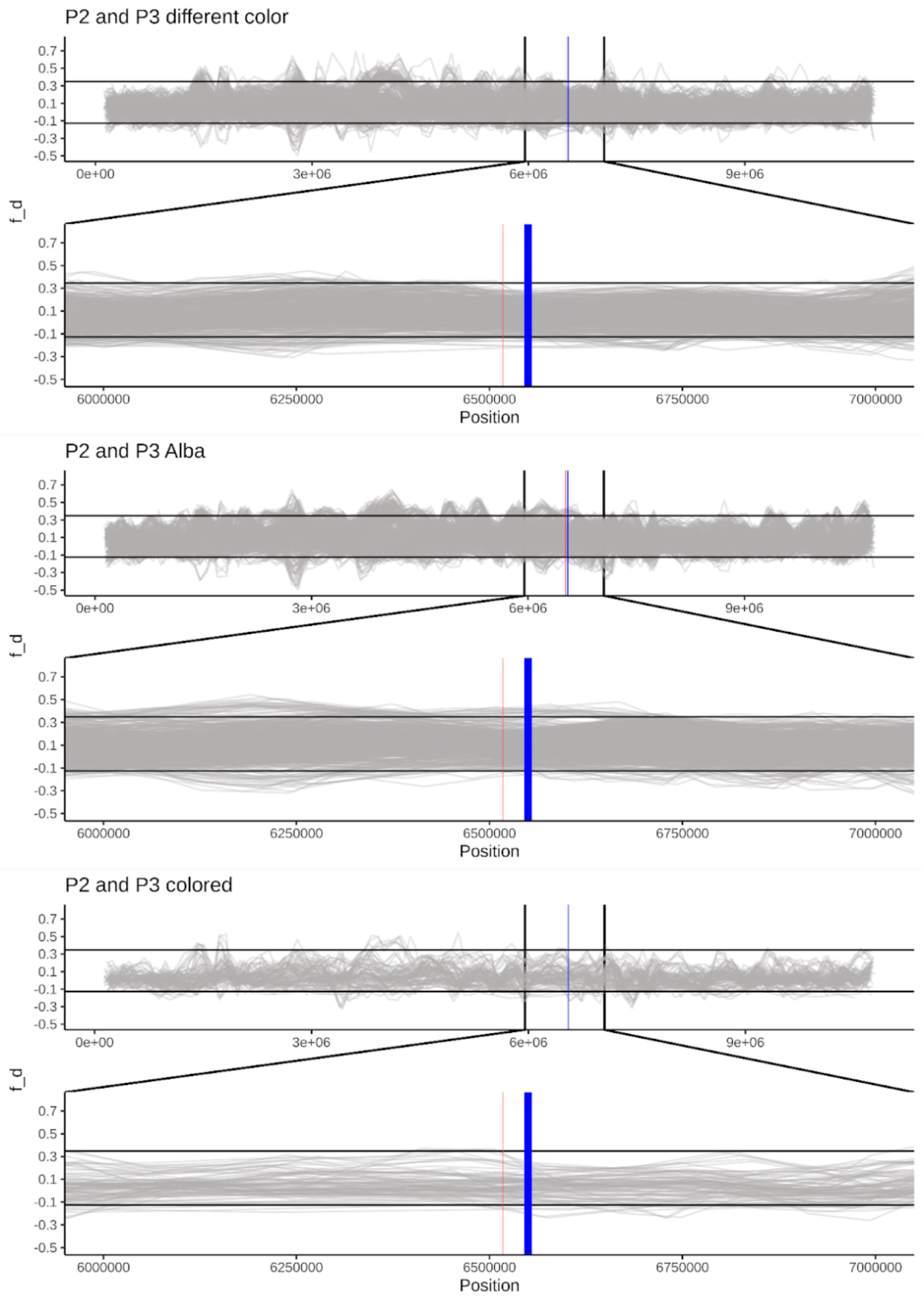

Fig. S5. Assessment of distance fraction (d_f) across scaffold 2 and the Alba locus in 500 bp sliding windows that overlap 250 bp. We included all trios that reported significant introgression in our Dmin analysis and grouped each trio based on the coloration of species P2 and P3 in each P1-P2-P3 species pair. *C. lesbia* was used as outgroup species P4 in all comparisons. The black horizontal lines represent the 95 percentile of the chromosome wide d_f of all comparisons. The BarH-1 gene is highlighted by a blue box and the *Alba* insertion with a red line. A highly positive or negative value of d_f is indicative of past introgression between P3 and P2 (positive) and P3 and P1 (negative). Notice how almost no trios show an increase in d_f around the *Alba* locus in trios where species P2 and P3 are either both orange or when they are of different colors, compared to when both are Alba, and we see many instances of high levels of introgression.

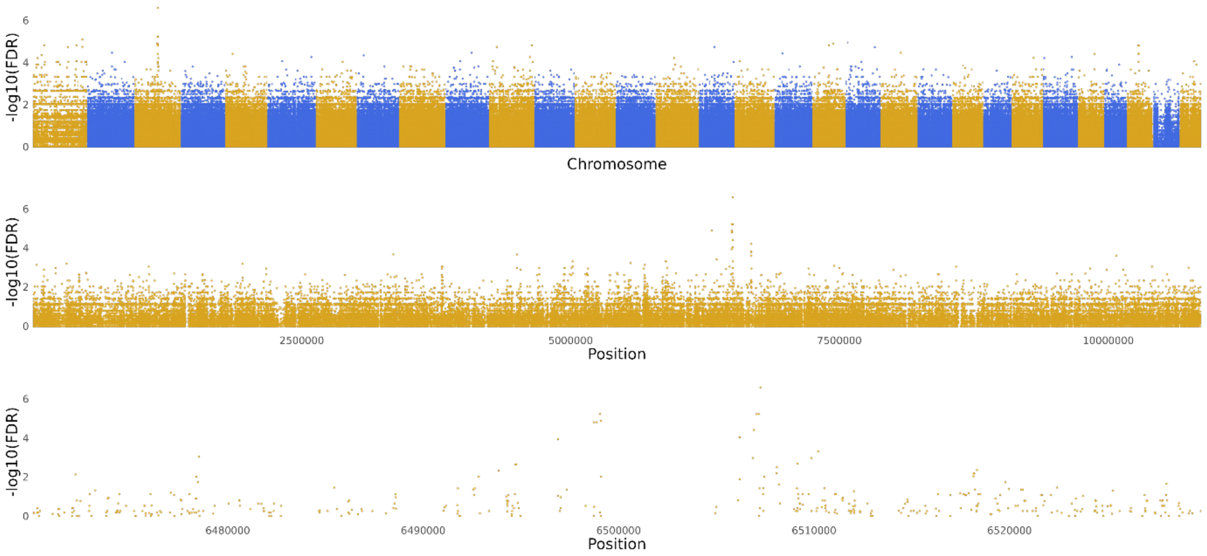

Fig. S6. Genome-wide association study using the orange *C. eurytheme* assembly as the reference genome. GWAS results against the orange reference genome using the light filters of quality and depth only. The Y-axis is log10(p-value) for SNPs correlating with Alba color. The X-axis represents genomic position. Top: Genome-wide results showing peak on Chromosome 3. Middle: Scaffold 2 within Chromosome 3. Bottom: A view of the 60kb around the highest peak.

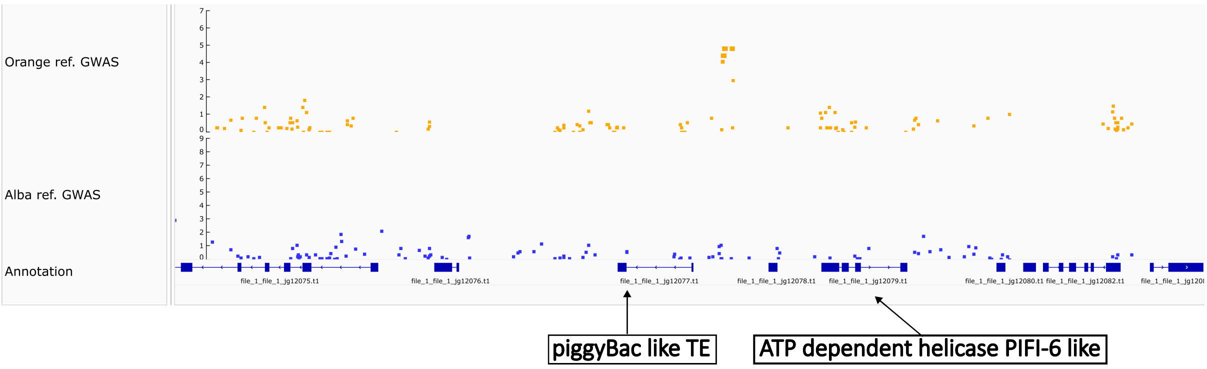

Fig. S7. Close up on the second GWAS locus identified on Scaffold 22 when the orange reference genome was used. The top row colored in orange represents sites identified against the orange reference genome, while the blue is from the synthetic Alba reference genome. The gene downstream of the highly associated loci is similar PIFI-like transposase when blasted against NCBI, and the gene directly upstream is similar to a PiggyBac transposon

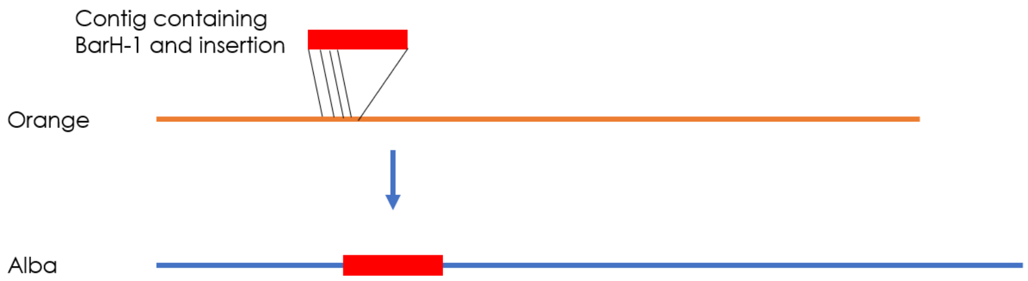

Fig. S8. Cartoon depicting construction of the *C. eurytheme* Alba reference genome. The contig containing BarH1 and the insertion was identified using blast and read depth analysis. Overlapping sequences between the contig and the orange reference genome were used to define the edges and to insert the sequence. The sequence was then inserted and overlapping sequences removed, favoring the Alba contig sequence leading to the generation of the Alba reference genome.

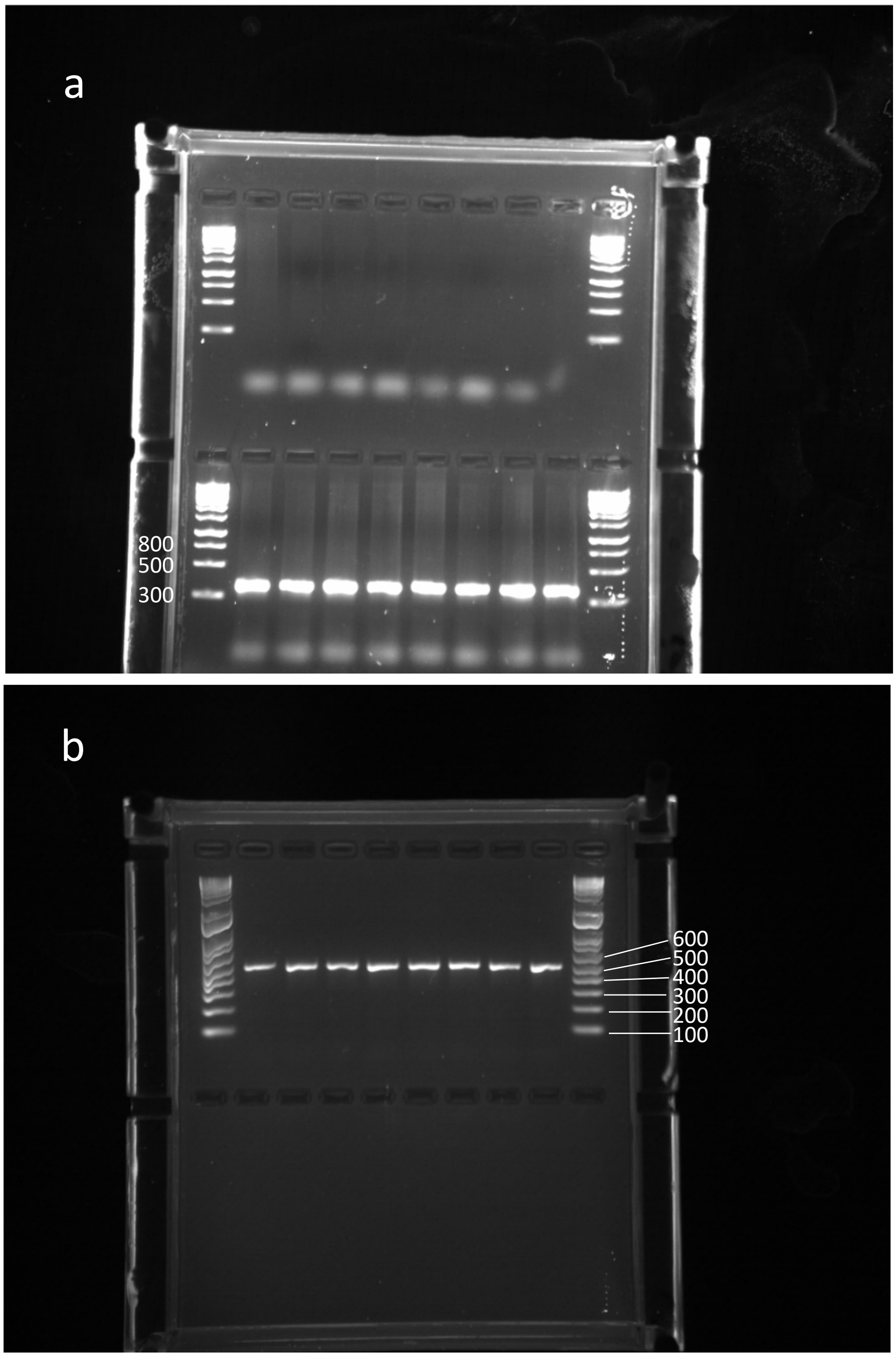

Fig. S9. Verification of the Alba locus in *C. eurytheme* using PCR-based markers. **a.** Eight orange and eight Alba *C. eurytheme* individuals, independent from the GWAS analysis, had DNA extracted and then genotyped for the insertion. PCR products were visualized on a 1% agarose gel. The top row shows negative results in orange females, and the bottom row shows positive results from Alba females. **b.** Positive control on orange female samples (from top row in panel in the same **a**.) using primers binding to mitochondrial cytochrome oxidase.

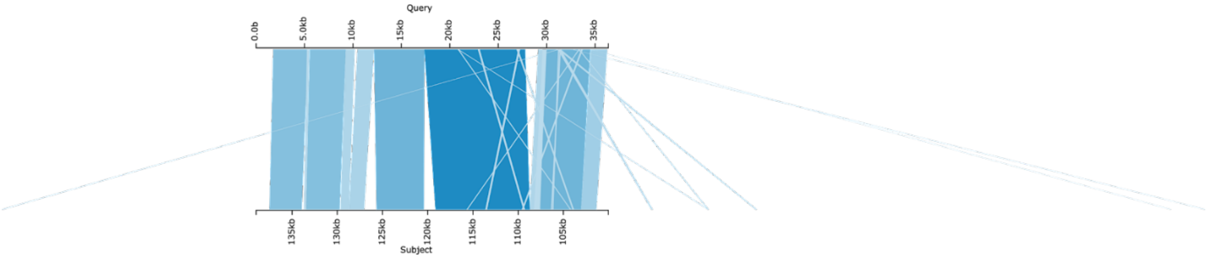

Fig. S10.
Orthology assessment of the *C. crocea Alba* scaffold identified in the 10X assembly (query), identified by using BlastN of the *C. eurytheme* BarH1 gene and insertion sequence against the assembly, compared to the one found in the *C. crocea* reference genome(subject). The darker the blue hue is, the higher identity is.

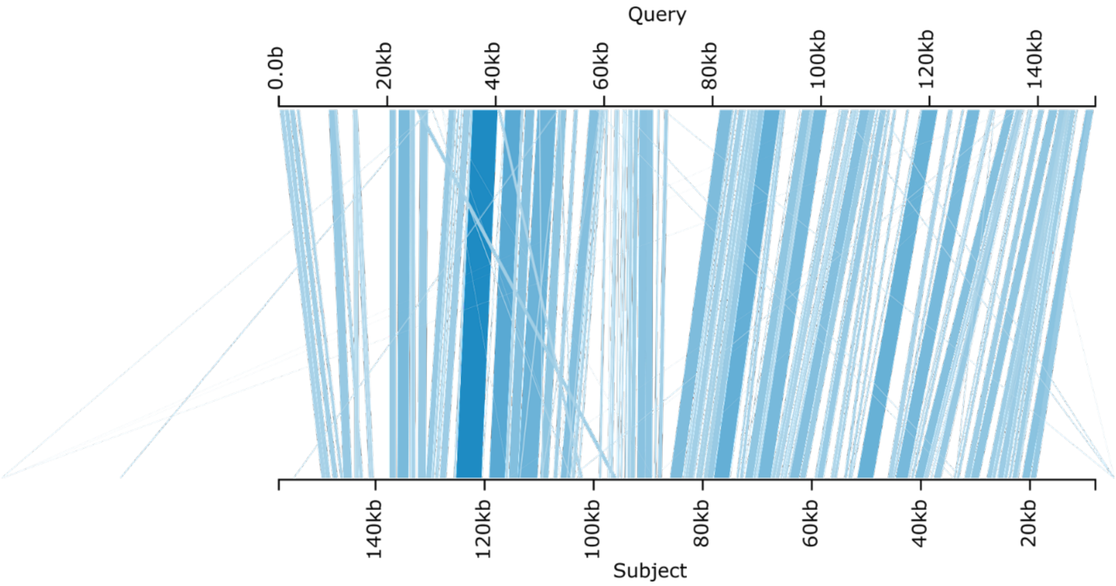

Fig. S11.
Orthology assessment of the Alba insertion in *C. nastes*. The Alba scaffold, as identified using the BarH-1 gene and insertion identified in *C. eurytheme*, from the *C. nastes* 10X assembly (query), aligned against the *Colias crocea* reference genome(subject). The darker the blue hue is, the higher identity is.

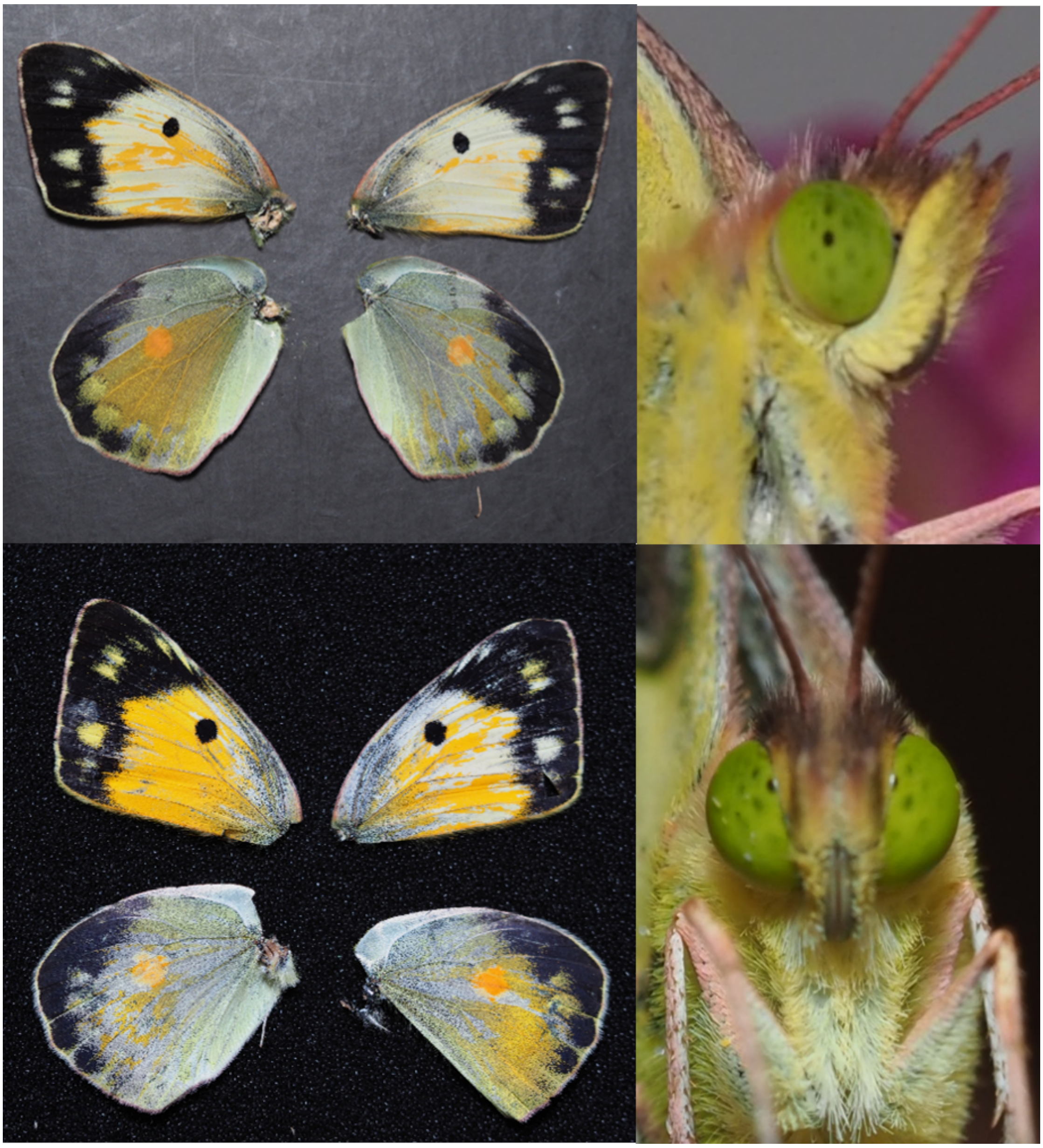

Fig. S12. *Alba* CRE knockout (KO). Images of both successful "conserved Alba region" CRISPR-KOs with their phenotypes: ind. Alba CRE-KO 1 wing (top left), ind. Alba CRE-KO 2 wing (bottom left), eye ind. Alba CRE-KO 1 (top right), eye ind. Alba CRE-KO 2 (bottom right)

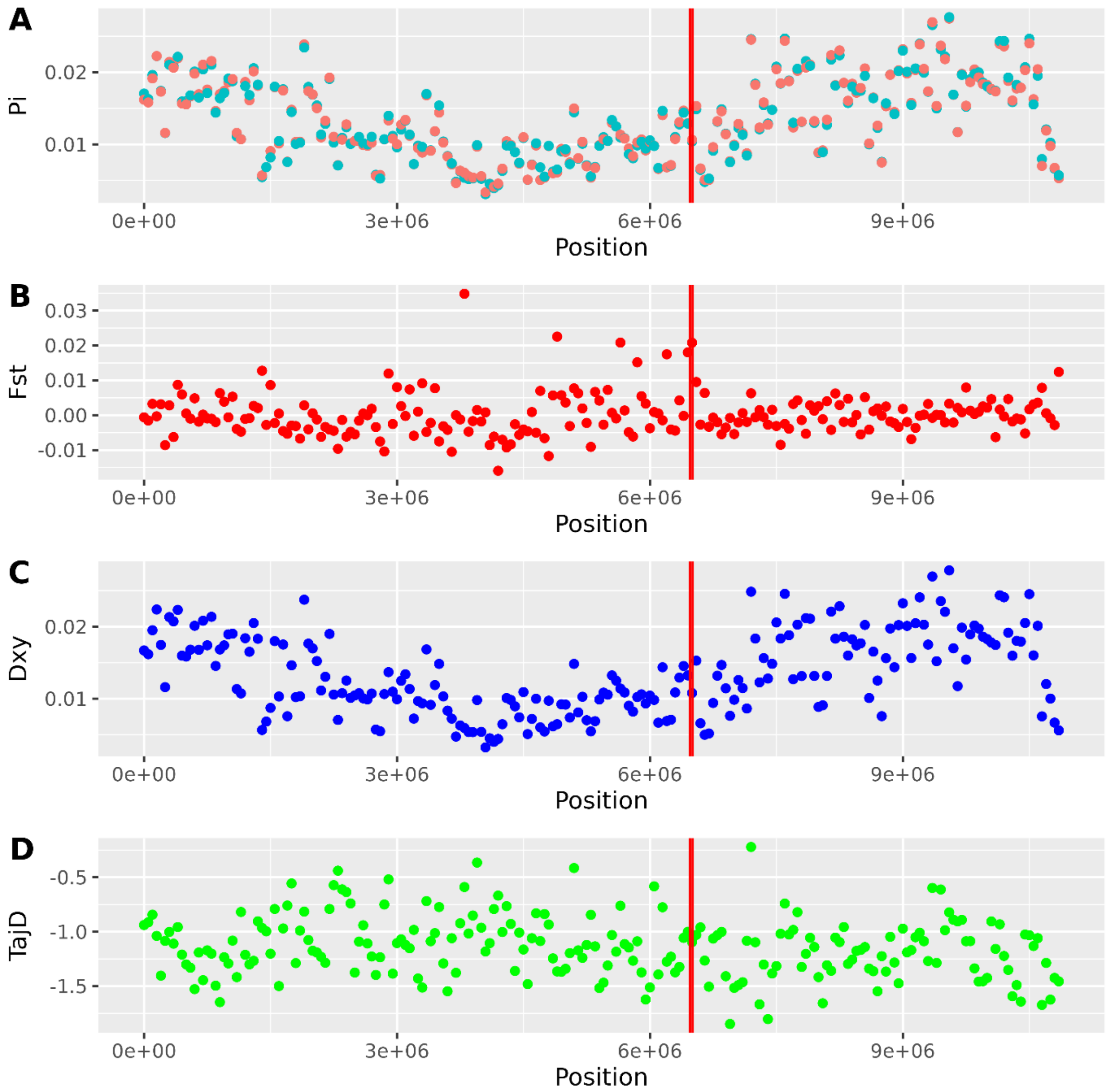

Fig. S13. Window based popgen analysis in *C. eurytheme.*
Window based scan for changes in **A.** nucleotide diversity (Pi), **B.** F_{st}, **C.** Dxy, and **D.** Tajima's D. Statistics in A-D were calculated in windows of 50kb. Pixy was used to generate **A.-C.** and vcftools for **D.** The red vertical bar represents the location of the Alba insertion. All scans were done using the orange reference genome as a reference.

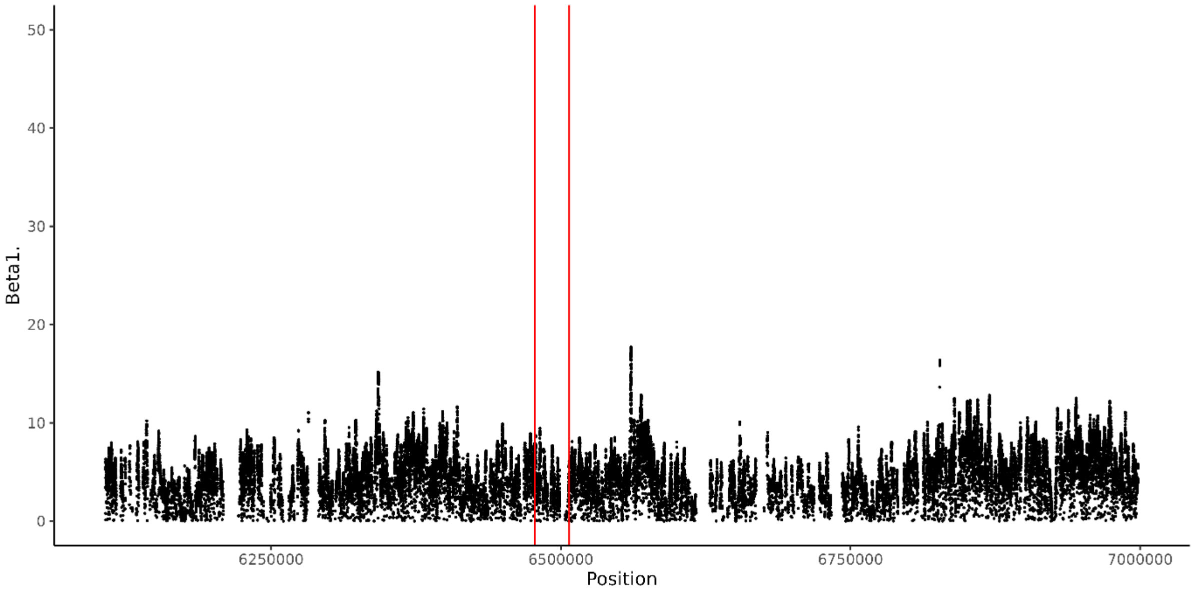

Fig. S14. Betascan analysis.
Beta statistics across the scaffold 02, on which the Alba locus is located in *C. eurytheme*, calculated against the orange reference genome. The red vertical lines represent the approximate region in which the *Alba* insertion is located.

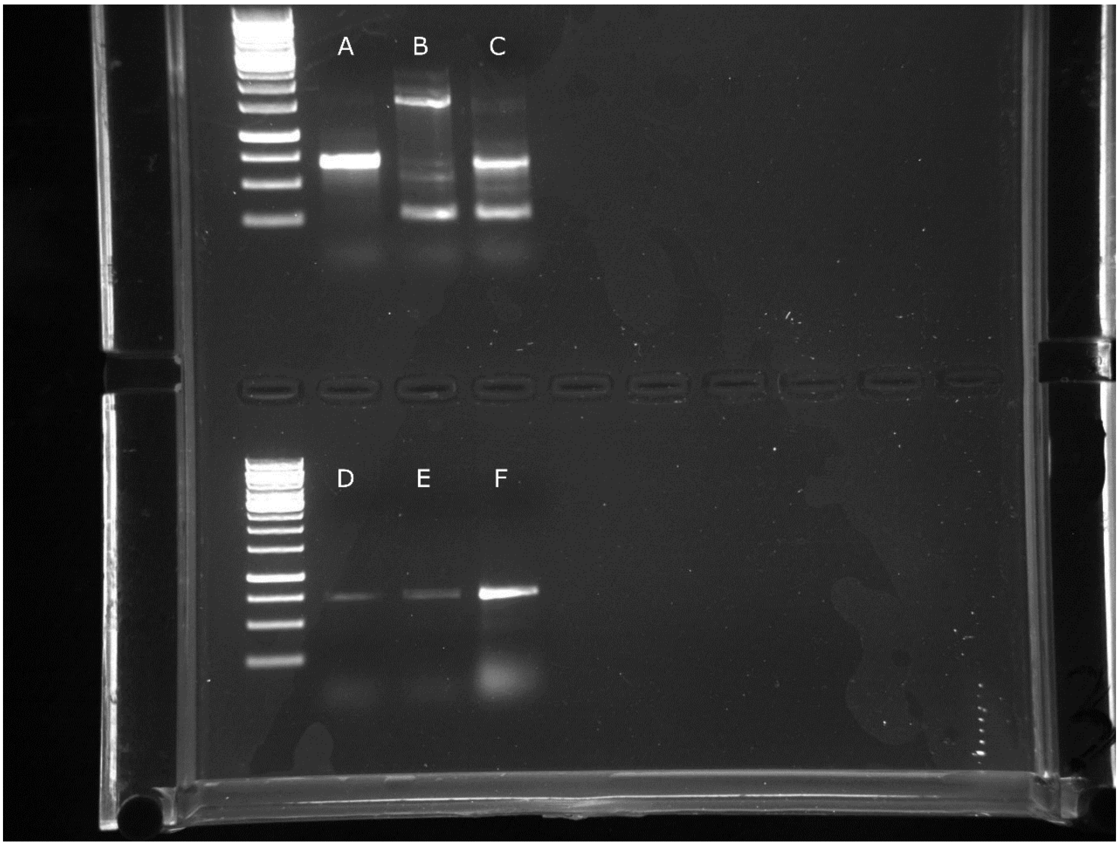

Fig. S15. Verification of CRISPR/Cas9 mutagenesis of the *Alba* candidate locus in *C. crocea* via PCR and gel electrophoresis. DNA was extracted from the thorax. Gel verifying CRISPR KO as well as Alba status for the two successful mutants. From left to right the wells show **A:** Alba-CRE WT; **B:** Alba-CRE KO-1; **C:** Alba-CRE KO-2; **D-F:** Alba control validation primer on the sample in the column above. Note the variation in band sizes between the two knock-out samples indicating deletion variation also seen in sequencing results of the deletion sites in Fig. S16.

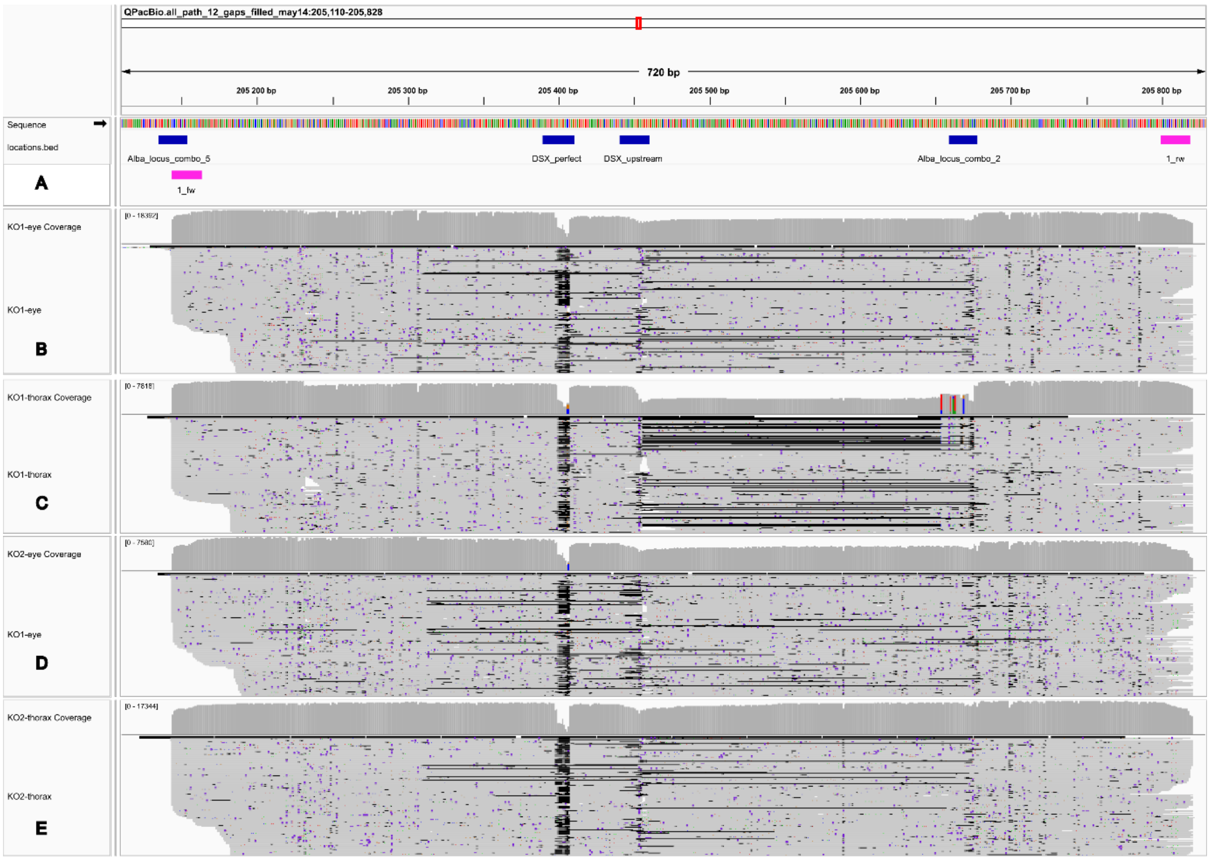

Fig. S16. Alba CRE KO cut site validation.

Pileup and coverage plot of Nanopore sequenced amplicons generated from the two successful KO-individuals. **A.** Location of gRNA cut sites (blue) and primer binding sites (red). The amplicons were amplified from DNA extracted from the eyes (**B, D**) and thorax (**C, E**) of the two successful knock out individuals. CRE-KO 1 is displayed in panel **B** and **C,** and CRE-KO-2 in panel **D** and **E.** Black lines indicate a deletion in the amplicon, and a gray means that it is matching the reference. The deletions overlap with locations of the gRNA target sites (with the exception of the rightmost gRNA that is missed by our primer pair. Deletions vary in size. Small deletions likely result from non-homologous end joining due to a single cut, while a larger deletion likely arises when 2 or more double stranded breaks occur due to multiple cut sites on the same DNA molecule. The gRNA that is causing a consistent deletion across both samples and tissues is located on top of the putative doublesex binding site.

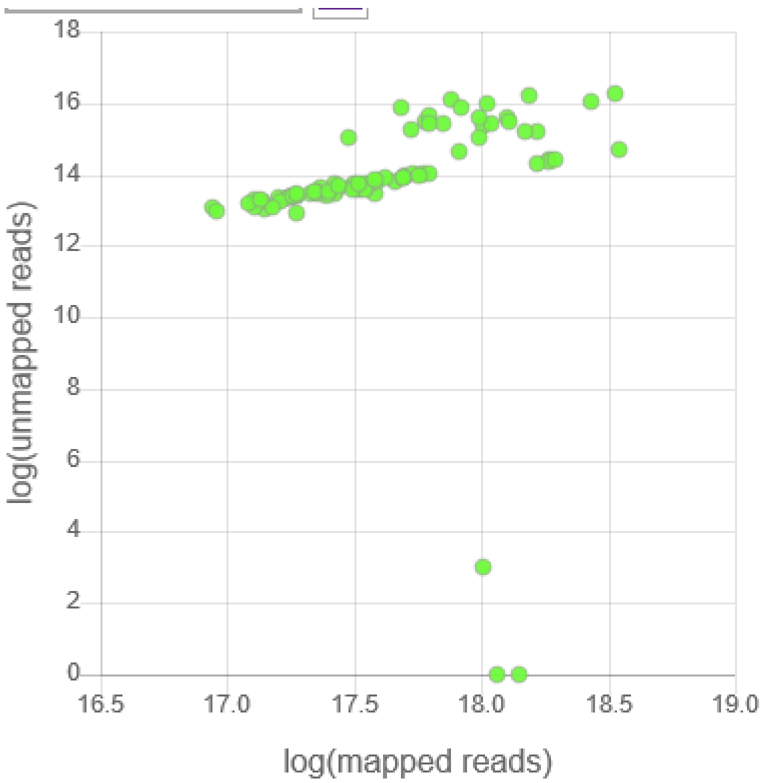

Fig. S17. Mappability of the all the short read datasets (all species, all samples). Scatterplot shows counts of mapped reads versus unmapped reads. Note that the samples with very few unmapped reads had been filtered for mapped reads prior to the generation of this figure.

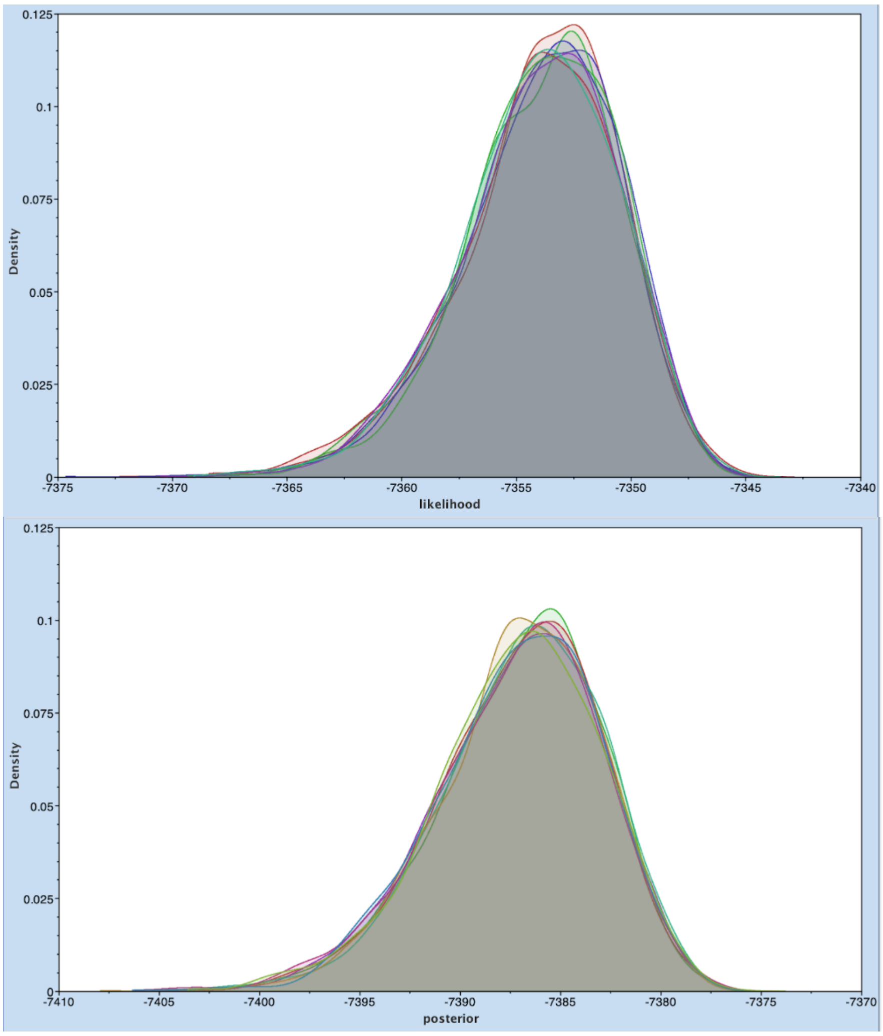

Fig. S18. SNAPP time calibration. Convergence assessment of independent SNAPP runs using different starting trees. Top panel is likelihood and bottom panel is posterior values for the final tree. Each run was for 4 million iterations, with 10% trimmed. For each plot, all 8 distributions are shown, where the differences in their overlapping regions are colored uniquely by run.

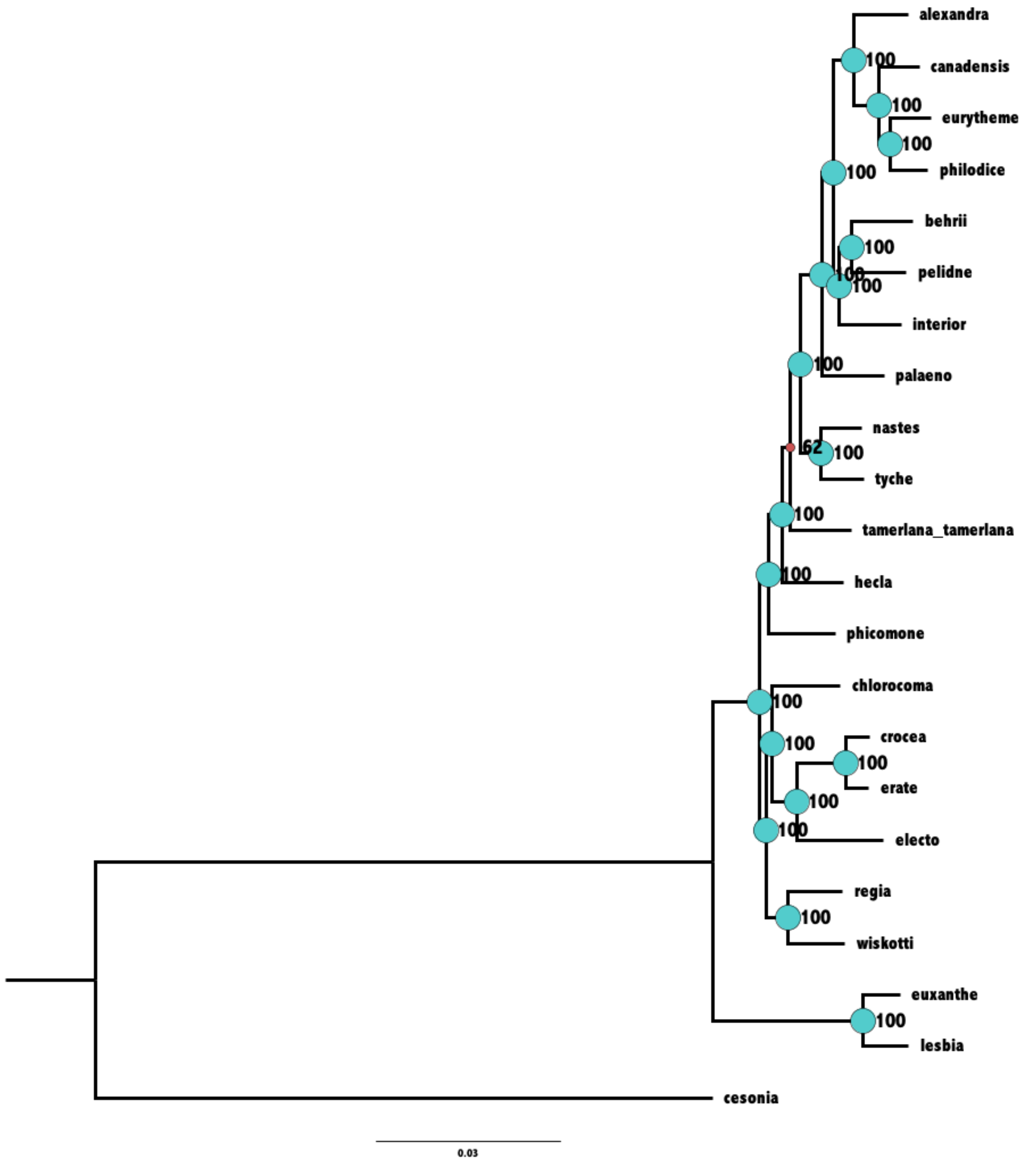

Fig. S19. Busco exon tree. Phylogenetic tree of the 21 taxa subset generated using concatenated busco_exons. This set of genes was used as input for iQtree. Tree is rooted with *Z. cesonia*, and for each node, the branch support is shown with values and color. Only one node has < 100 % bootstrap support.

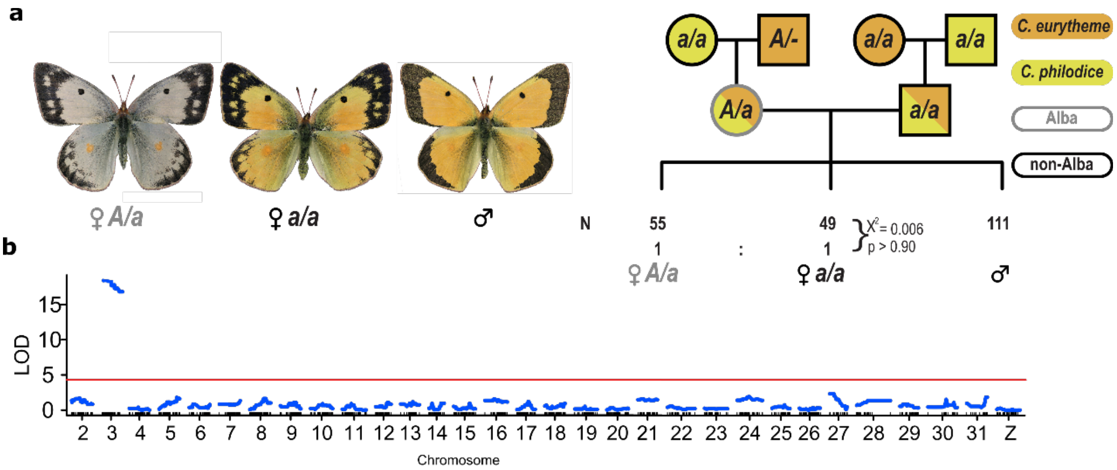

Fig. S20. QTL mapping and GWAS of Alba in *C. eurytheme*.

a. *Colias eurytheme* specimens and schematic of female informative crosses used in the linkage analysis. **b.** Linkage mapping of the Alba trait generated from female informative hybrid crosses of *C. eurytheme* and *C. philodice* revealed a single autosomal locus on Chr. 3 that was associated with Alba.

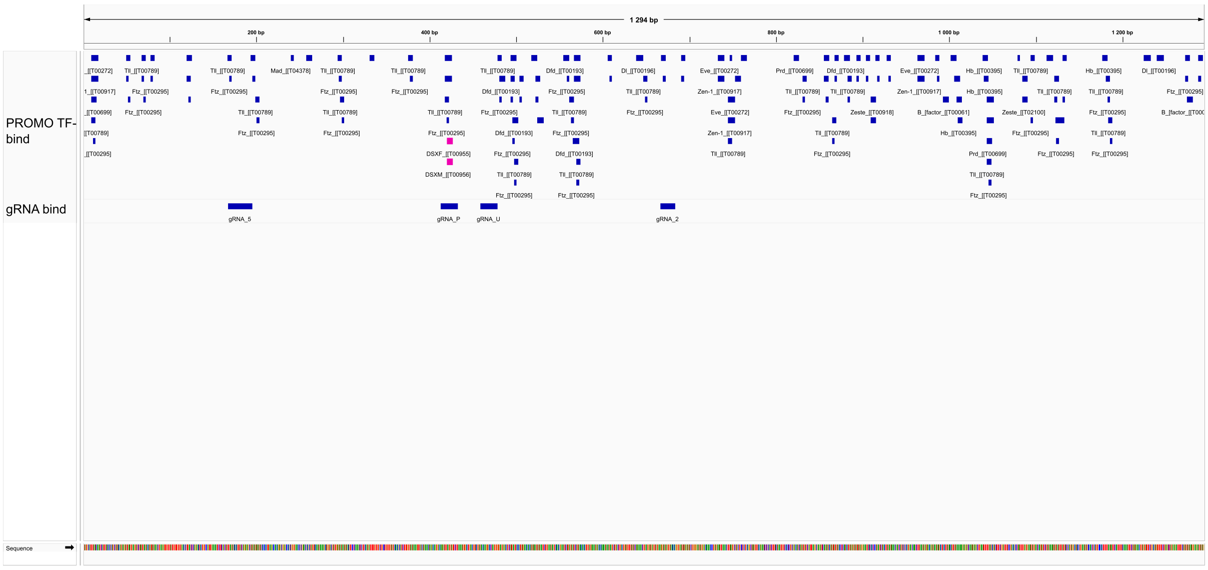

Fig. S21. Predicted transcription factor binding sites in Alba candidate locus.
Top track is showing the predicted transcription factor binding sites according to PROMO within the *Alba* candidate locus. The lower track indicates the position of the guide RNAs used, with the two middle ones located nearest the location of the doublesex binding site (highlighted in pink). See Table S9 for more details.

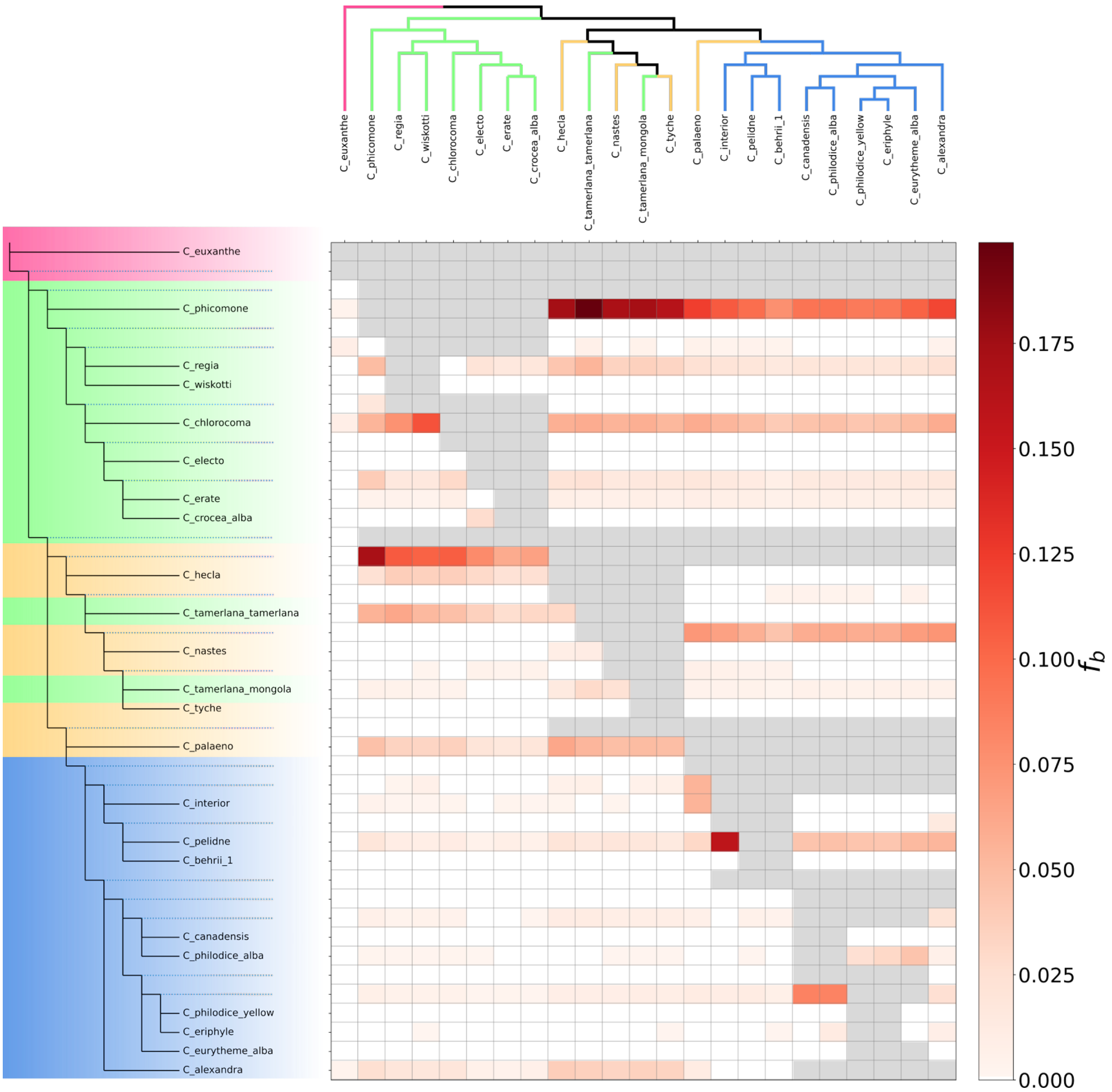

Fig. S22. Signatures of historical introgression across the *Colias* species tree phylogeny. Each cell in the grid indicates the f-branch statistic, identifying excess sharing of derived alleles between branch nodes on the y-axis (blue dotted lines) and individual species on the x-axis. A darker color in the heatmap indicates higher f_b, suggesting gene flow between that branch and species. Results indicate a strong signal of introgression between an ancestor of the *C. nastes* clade and the Eurasian species and between *C. phicomone* and the North American species. Species and internal nodes are colored by the species' current distribution where purple = South America, blue = North America, orange = Holarctic, green = Eurasia and Northern Africa.

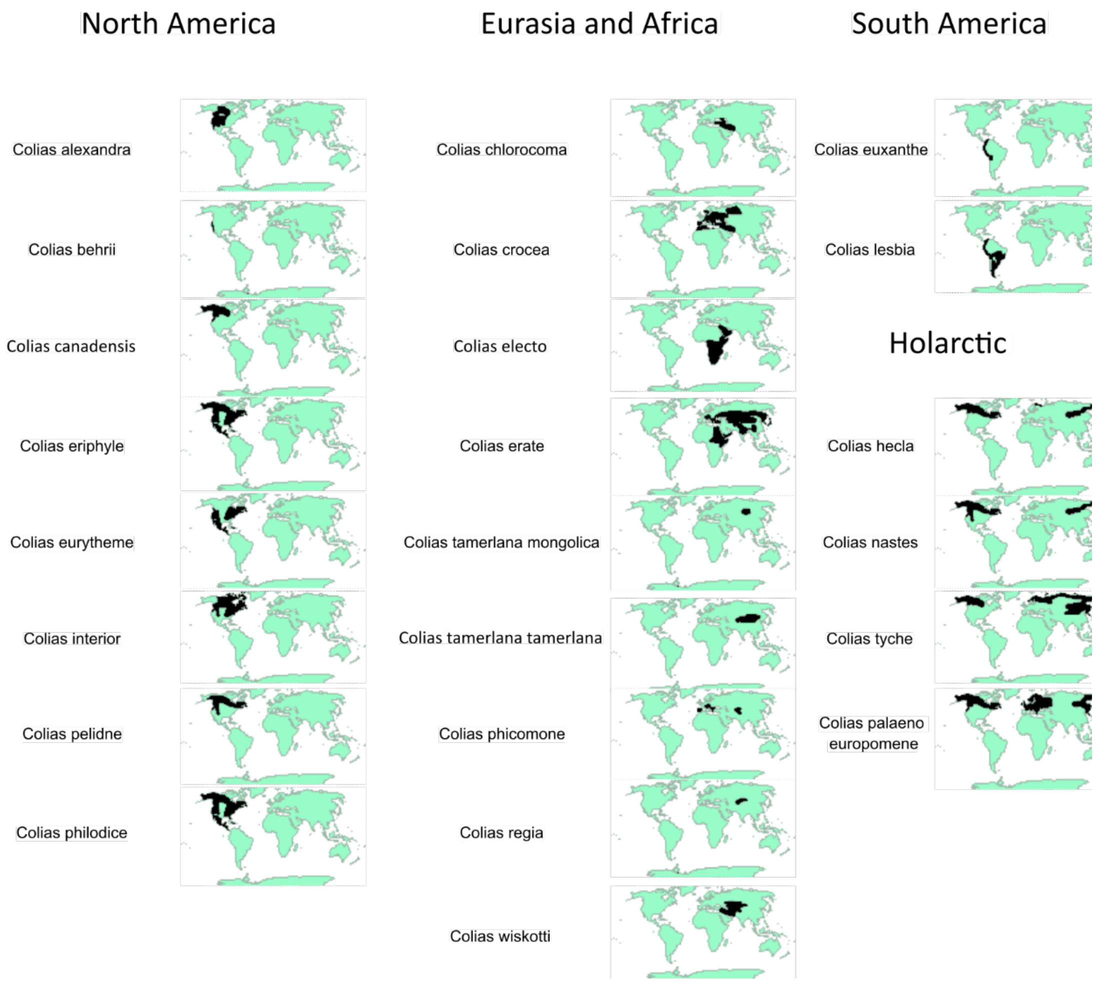

Fig. S23.

Distribution of species used in our phylogenetic analysis. This information was not used in our analyses and are only included to facilitate general insights into approximate ranges. The locations and maps are all taken from the funet website. These were automatically generated from regional information drawn from primary literature using a text mining approach. As such, these maps are approximate and included here for a general overview. However, should readers wish to have more accurate, more confident range information, we direct them to the funet website and the primary literature listed therein.

Colias: https://www.funet.fi/pub/sci/bio/life/insecta/lepidoptera/ditrysia/papilionoidea/pieridae/coliadinae/colias/index.html

About range maps: https://www.nic.funet.fi/pub/sci/bio/life/about-maps.html

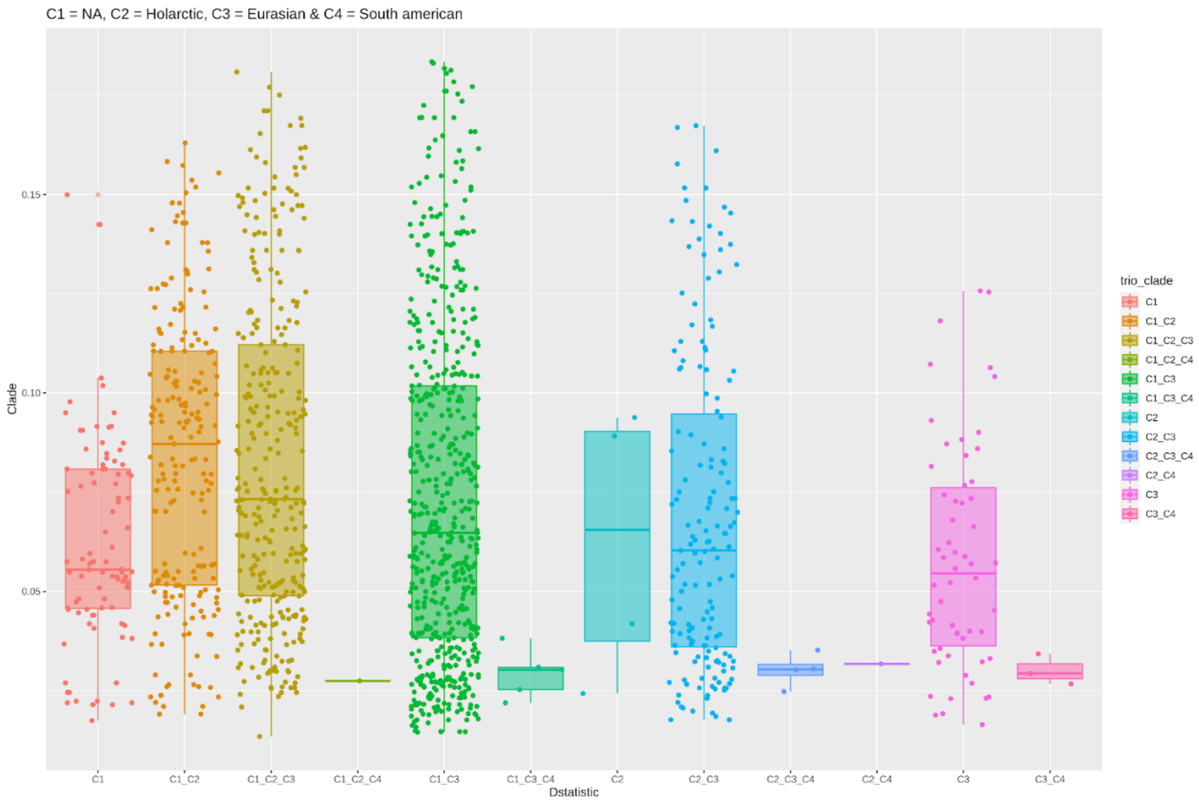

Fig. S24. Distribution of minimal D-statistic of all species-trios that showed significant levels of introgression (Bonferroni-Holm corrected $p < 0.05$). Trios are grouped by the geographic composition of the trio.

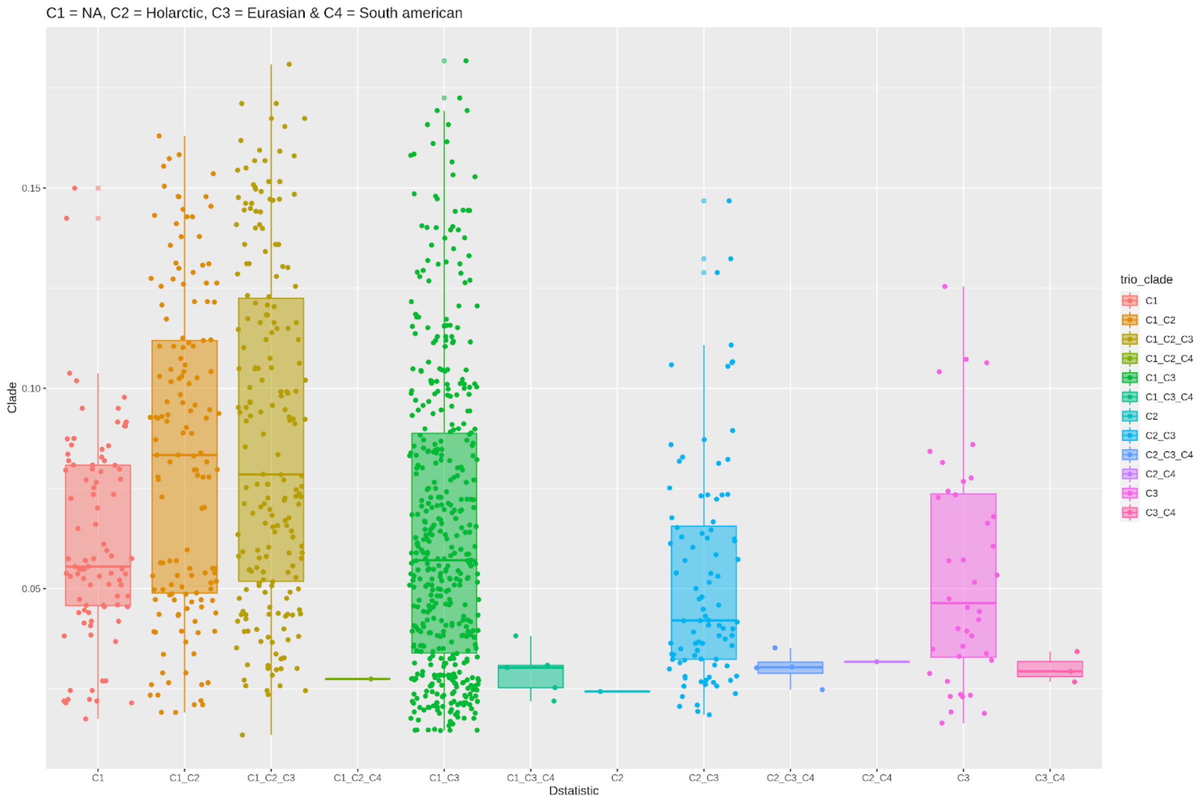

Fig. S25. Distribution of minimal D-statistic of all species-trios that showed significant levels of introgression after removing any trio that contained *C. phicomone* or *C. palaeno* (Bonferroni-Holm corrected $p < 0.05$). Trios are grouped by the geographic composition of the trio.

REFERENCES

1. M. R. Rose, L. D. Mueller, Stearns, Stephen C., 1992. The Evolution of Life Histories. Oxford University Press, London xii + 249 pp., f16.95. *J. Evol. Biol.* **6**, 304–306 (1993).

2. T. Flatt, A. Heyland, *Mechanisms of Life History Evolution: The Genetics and Physiology of Life History Traits and Trade-offs* (Oxford Univ. Press, 2011).

3. M. R. Gross, Alternative reproductive strategies and tactics: Diversity within sexes. *Trends Ecol. Evol.* **11**, 92–98 (1996).

4. M. J. West-Eberhard, *Developmental Plasticity and Evolution* (Oxford Univ. Press, 2003).

5. G. A. Jamie, J. I. Meier, The persistence of polymorphisms across species radiations. *Trends Ecol. Evol.* **35**, 795–808 (2020).

6. E. B. Ford, Polymorphism. *Biol. Rev.* **20**, 73–88 (1945).

7. V. Llaurens, A. Whibley, M. Joron, Genetic architecture and balancing selection: The life and death of differentiated variants. *Mol. Ecol.* **26**, 2430–2448 (2017).

8. C. Mérot, V. Llaurens, E. Normandeau, L. Bernatchez, M. Wellenreuther, Balancing selection via life-history trade-offs maintains an inversion polymorphism in a seaweed fly. *Nat. Commun.* **11**, 670 (2020).

9. A. Yassin, E. K. Delaney, A. J. Reddiex, T. D. Seher, H. Bastide, N. C. Appleton, J. B. Lack, J. R. David, S. F. Chenoweth, J. E. Pool, A. Kopp, The *pdm3* locus is a hotspot for recurrent evolution of female-limited color dimorphism in *Drosophila*. *Curr. Biol.* **26**, 2412–2422 (2016).

10. R. Blow, B. Willink, E. I. Svensson, A molecular phylogeny of forktail damselflies (genus *Ischnura*) reveals a dynamic macroevolutionary history of female colour polymorphisms. *Mol. Phylogenet. Evol.* **160**, 107134 (2021).

11. C. Grossen, L. Keller, I. Biebach; The International Goat Genome Consortium, D. Croll, Introgression from domestic goat generated variation at the major histocompatibility complex of alpine ibex. *PLOS Genet.* **10**, e1004438 (2014).

12. E. M. Tuttle, A. O. Bergland, M. L. Korody, M. S. Brewer, D. J. Newhouse, P. Minx, M. Stager, A. Betuel, Z. A. Cheviron, W. C. Warren, R. A. Gonser, C. N. Balakrishnan, Divergence and functional degradation of a sex chromosome-like supergene. *Curr. Biol.* **26**, 344–350 (2016).

13. D. H. Palmer, M. R. Kronforst, A shared genetic basis of mimicry across swallowtail butterflies points to ancestral co-option of doublesex. *Nat. Commun.* **11**, 6 (2020).

14. R. W. R. Wallbank, S. W. Baxter, C. Pardo-Diaz, J. J. Hanly, S. H. Martin, J. Mallet, K. K. Dasmahapatra, C. Salazar, M. Joron, N. Nadeau, W. O. McMillan, C. D. Jiggins, Evolutionary novelty in a butterfly wing pattern through enhancer shuffling. *PLOS Biol.* **14**, e1002353 (2016).

15. E. L. Westerman, R. Letchinger, A. Tenger-Trolander, D. Massardo, D. Palmer, M. R. Kronforst, Does male preference play a role in maintaining female limited polymorphism in a Batesian mimetic butterfly? *Behav. Processes* **150**, 47–58 (2018).

16. J. Morris, J. J. Hanly, S. H. Martin, S. M. V. Belleghem, C. Salazar, C. D. Jiggins, K. K. Dasmahapatra, Deep convergence, shared ancestry, and evolutionary novelty in the genetic architecture of *Heliconius* mimicry. *Genetics* **216**, 765–780 (2020).

17. J. J. Lewis, R. C. Geltman, P. C. Pollak, K. E. Rondem, S. M. V. Belleghem, M. J. Hubisz, P. R. Munn, L. Zhang, C. Benson, A. Mazo-Vargas, C. G. Danko, B. A. Counterman, R. Papa, R. D. Reed, Parallel evolution of ancient, pleiotropic enhancers underlies butterfly wing pattern mimicry. *Proc. Natl. Acad. Sci. U.S.A.* **116**, 24174–24183 (2019).

18. J. J. Lewis, S. M. Van Belleghem, R. Papa, C. G. Danko, R. D. Reed, Many functionally connected loci foster adaptive diversification along a neotropical hybrid zone. *Sci. Adv.* **6**, eabb8617 (2020).

19. J. J. Lewis, S. M. Van Belleghem, Mechanisms of change: A population-based perspective on the roles of modularity and pleiotropy in diversification. *Front. Ecol. Evol.* **8**, 261 (2020).

20. C. L. Remington, The genetics of Colias (Lepidoptera), in *Advances in Genetics*, M. Demerec, Ed. (Academic Press, 1954), vol. 6, pp. 403–450.

21. L. B. Limeri, N. I. Morehouse, The evolutionary history of the 'alba' polymorphism in the butterfly subfamily Coliadinae (Lepidoptera: Pieridae). *Biol. J. Linn. Soc.* **117**, 716–724 (2016).

22. W. B. Watt, Adaptive significance of pigment polymorphisms in Colias butterflies. III. Progress in the study of the "Alba" variant. *Evolution* **27**, 537–548 (1973).

23. S. M. Graham, W. B. Watt, L. F. Gall, Metabolic resource allocation vs. mating attractiveness: Adaptive pressures on the "alba" polymorphism of *Colias* butterflies. *Proc. Natl. Acad. Sci. U.S.A.* **77**, 3615–3619 (1980).

24. H. Descimon, J.-L. Pennetier, Nitrogen metabolism in *Colias croceus* (Linné) and its "*Alba*" mutant (Lepidoptera Pieridae). *J. Insect Physiol.* **35**, 881–885 (1989).

25. M. G. Nielsen, W. B. Watt, Behavioural fitness component effects of the alba polymorphism of *Colias* (Lepidoptera, Pieridae): Resource and time budget analysis. *Funct. Ecol.* **12**, 149–158 (1998).

26. A. Woronik, C. Stefanescu, R. Käkelä, C. W. Wheat, P. Lehmann, Physiological differences between female limited, alternative life history strategies: The Alba phenotype in the butterfly Colias croceus. *J. Insect Physiol.* **107**, 257–264 (2018).

27. G. W. Gilchrist, R. L. Rutowski, Adaptive and incidental consequences of the alba polymorphism in an agricultural population of Colias butterflies: Female size, fecundity, and differential dispersion. *Oecologia* **68**, 235–240 (1986).

28. A. Woronik, K. Tunström, M. W. Perry, R. Neethiraj, C. Stefanescu, M. de la P. Celorio-Mancera, O. Brattström, J. Hill, P. Lehmann, R. Käkelä, C. W. Wheat, A transposable

element insertion is associated with an alternative life history strategy. *Nat. Commun.* **10**, 5757 (2019).

29. W. Hovanitz, The biology of Colias butterfly II. Parallel geographical variation of dimorphic color phases in north America species. *Wassman J. Biol.* **8**, 197–219 (1950).

30. M. G. Nielsen, W. B. Watt, Interference competition and sexual selection promote polymorphism in Colias (Lepidoptera, Pieridae). *Funct. Ecol.* **14**, 718–730 (2000).

31. C. W. Wheat, W. B. Watt, A mitochondrial-DNA-based phylogeny for some evolutionary-genetic model species of Colias butterflies (Lepidoptera, Pieridae). *Mol. Phylogenet. Evol.* **47**, 893–902 (2008).

32. N. Chazot, N. Wahlberg, A. V. L. Freitas, C. Mitter, C. Labandeira, J.-C. Sohn, R. K. Sahoo, N. Seraphim, R. de Jong, M. Heikkilä, Priors and posteriors in Bayesian timing of divergence analyses: The age of butterflies revisited. *Syst. Biol.* **68**, 797–813 (2019).

33. M. Stange, M. R. Sánchez-Villagra, W. Salzburger, M. Matschiner, Bayesian divergence-time estimation with genome-wide single-nucleotide polymorphism data of sea catfishes (Ariidae) supports Miocene closure of the Panamanian Isthmus. *Syst. Biol.* **67**, 681–699 (2018).

34. H. Descimon, J. Mallet, Bad species. *Ecol. Butterflies Eur.*, 219–249 (2009).

35. M. Malinsky, H. Svardal, A. M. Tyers, E. A. Miska, M. J. Genner, G. F. Turner, R. Durbin, Whole-genome sequences of Malawi cichlids reveal multiple radiations interconnected by gene flow. *Nat. Ecol. Evol.* **2**, 1940–1955 (2018).

36. J. A. Scott, *The Butterflies of North America: A Natural History and Field Guide* (Stanford Univ. Press, 1992).

37. L. A. Berger, *Systématique du genre Colias F.: Lepidoptera-Pieridae (222)* (Imprimerie des Sciences, 1986).

38. W. Hovanitz, The ecological significance of the color phases of Colias chrysotheme in North America. *Ecology* **25**, 45–60 (1944).

39. J. L. Feder, X. Xie, J. Rull, S. Velez, A. Forbes, B. Leung, H. Dambroski, K. E. Filchak, M. Aluja, Mayr, Dobzhansky, and Bush and the complexities of sympatric speciation in *Rhagoletis*. *Proc. Natl. Acad. Sci. U.S.A.* **102**, 6573–6580 (2005).

40. B. Pfeifer, D. D. Kapan, Estimates of introgression as a function of pairwise distances. *BMC Bioinformatics* **20**, 207 (2019).

41. A. Fijarczyk, W. Babik, Detecting balancing selection in genomes: Limits and prospects. *Mol. Ecol.* **24**, 3529–3545 (2015).

42. F. Tajima, Statistical method for testing the neutral mutation hypothesis by DNA polymorphism. *Genetics* **123**, 585–595 (1989).

43. K. M. Siewert, B. F. Voight, BetaScan2: Standardized statistics to detect balancing selection utilizing substitution data. *Genome Biol. Evol.* **12**, 3873–3877 (2020).

44. W. Hovanitz, The distribution of gene frequencies in wild populations of Colias. *Genetics* **29**, 31–60 (1944).

45. G. A. Wray, The evolutionary significance of cis-regulatory mutations. *Nat. Rev. Genet.* **8**, 206–216 (2007).

46. W. Hovanitz, The distribution of Colias in the equatorial Andes. *Caldasia* **3**, 283–300 (1945).

47. The Heliconius Genome Consortium, K. K. Dasmahapatra, J. R. Walters, A. D. Briscoe, J. W. Davey, A. Whibley, N. J. Nadeau, A. V. Zimin, D. S. T. Hughes, L. C. Ferguson, S. H. Martin, C. Salazar, J. J. Lewis, S. Adler, S.-J. Ahn, D. A. Baker, S. W. Baxter, N. L. Chamberlain, R. Chauhan, B. A. Counterman, T. Dalmay, L. E. Gilbert, K. Gordon, D. G. Heckel, H. M. Hines, K. J. Hoff, P. W. H. Holland, E. Jacquin-Joly, F. M. Jiggins, R. T. Jones, D. D. Kapan, P. Kersey, G. Lamas, D. Lawson, D. Mapleson, L. S. Maroja, A. Martin, S. Moxon, W. J. Palmer, R. Papa, A. Papanicolaou, Y. Pauchet, D. A. Ray, N. Rosser, S. L.

Salzberg, M. A. Supple, A. Surridge, A. Tenger-Trolander, H. Vogel, P. A. Wilkinson, D. Wilson, J. A. Yorke, F. Yuan, A. L. Balmuth, C. Eland, K. Gharbi, M. Thomson, R. A. Gibbs, Y. Han, J. C. Jayaseelan, C. Kovar, T. Mathew, D. M. Muzny, F. Ongeri, L.-L. Pu, J. Qu, R. L. Thornton, K. C. Worley, Y.-Q. Wu, M. Linares, M. L. Blaxter, R. H. French-Constant, M. Joron, M. R. Kronforst, S. P. Mullen, R. D. Reed, S. E. Scherer, S. Richards, J. Mallet, W. Owen McMillan, C. D. Jiggins, Butterfly genome reveals promiscuous exchange of mimicry adaptations among species. *Nature* **487**, 94–98 (2012).

48. T. Hayashi, T. Kojima, K. Saigo, Specification of primary pigment cell and outer photoreceptor fates by BarH1 homeobox gene in the developing Drosophila eye. *Dev. Biol.* **200**, 131–145 (1998).

49. T. Kojima, M. Sato, K. Saigo, Formation and specification of distal leg segments in Drosophila by dual Bar homeobox genes, BarH1 and BarH2. *Development* **127**, 769–778 (2000).

50. G. Reig, M. E. Cabrejos, M. L. Concha, Functions of BarH transcription factors during embryonic development. *Dev. Biol.* **302**, 367–375 (2007).

51. D. A. Ernst, E. L. Westerman, Stage- and sex-specific transcriptome analyses reveal distinctive sensory gene expression patterns in a butterfly. *BMC Genomics* **22**, 584 (2021).

52. D.-Z. Li, S.-G. Duan, R.-N. Yang, S.-C. Yi, A. Liu, H. E. Abdelnabby, M.-Q. Wang, BarH1 regulates odorant-binding proteins expression and olfactory perception of Monochamus alternatus Hope. *Insect Biochem. Mol. Biol.* **140**, 103677 (2022).

53. M. Pavlicev, G. P. Wagner, A model of developmental evolution: Selection, pleiotropy and compensation. *Trends Ecol. Evol.* **27**, 316–322 (2012).

54. B. Prud'homme, N. Gompel, S. B. Carroll, Emerging principles of regulatory evolution. *Proc. Natl. Acad. Sci. U.S.A.* **104**, 8605–8612 (2007).

55. C.-S. Chin, P. Peluso, F. J. Sedlazeck, M. Nattestad, G. T. Concepcion, A. Clum, C. Dunn, R. O'Malley, R. Figueroa-Balderas, A. Morales-Cruz, G. R. Cramer, M. Delledonne, C. Luo, J.

R. Ecker, D. Cantu, D. R. Rank, M. C. Schatz, Phased diploid genome assembly with single-molecule real-time sequencing. *Nat. Methods* **13**, 1050–1054 (2016).

56. S. Huang, M. Kang, A. Xu, HaploMerger2: Rebuilding both haploid sub-assemblies from high-heterozygosity diploid genome assembly. *Bioinformatics* **33**, 2577–2579 (2017).

57. B. J. Walker, T. Abeel, T. Shea, M. Priest, A. Abouelliel, S. Sakthikumar, C. A. Cuomo, Q. Zeng, J. Wortman, S. K. Young, A. M. Earl, Pilon: An integrated tool for comprehensive microbial variant detection and genome assembly improvement. *PLOS ONE* **9**, e112963 (2014).

58. F. J. Sedlazeck, P. Rescheneder, A. von Haeseler, NextGenMap: Fast and accurate read mapping in highly polymorphic genomes. *Bioinformatics* **29**, 2790–2791 (2013).

59. F. A. Simão, R. M. Waterhouse, P. Ioannidis, E. V. Kriventseva, E. M. Zdobnov, BUSCO: Assessing genome assembly and annotation completeness with single-copy orthologs. *Bioinformatics* **31**, 3210–3212 (2015).

60. M. Seppey, M. Manni, E. M. Zdobnov, BUSCO: Assessing genome assembly and annotation completeness. *Methods Mol. Biol.* **1962**, 227–245 (2019).

61. H. Z. Girgis, Red: An intelligent, rapid, accurate tool for detecting repeats de-novo on the genomic scale. *BMC Bioinform.* **16**, 227 (2015).

62. T. Brůna, K. J. Hoff, A. Lomsadze, M. Stanke, M. Borodovsky, BRAKER2: Automatic eukaryotic genome annotation with GeneMark-EP+ and AUGUSTUS supported by a protein database. *NAR Genom. Bioinform.* **3**, lqaa108 (2021).

63. S. Nallu, J. A. Hill, K. Don, C. Sahagun, W. Zhang, C. Meslin, E. Snell-Rood, N. L. Clark, N. I. Morehouse, J. Bergelson, C. W. Wheat, M. R. Kronforst, The molecular genetic basis of herbivory between butterflies and their host plants. *Nat. Ecol. Evol.* **2**, 1418–1427 (2018).

64. D. Kim, J. M. Paggi, C. Park, C. Bennett, S. L. Salzberg, Graph-based genome alignment and genotyping with HISAT2 and HISAT-genotype. *Nat. Biotechnol.* **37**, 907–915 (2019).

65. L. Rodriguez-Caro, J. Fenner, C. Benson, S. M. Van Belleghem, B. A. Counterman, Genome assembly of the Dogface butterfly Zerene cesonia. *Genome Biol. Evol.* **12**, 3580–3585 (2020).

66. G. Marçais, A. L. Delcher, A. M. Phillippy, R. Coston, S. L. Salzberg, A. Zimin, MUMmer4: A fast and versatile genome alignment system. *PLOS Comput. Biol.* **14**, e1005944 (2018).

67. Z. Gu, L. Gu, R. Eils, M. Schlesner, B. Brors, *circlize* implements and enhances circular visualization in R. *Bioinformatics* **30**, 2811–2812 (2014).

68. S. M. Aljanabi, I. Martinez, Universal and rapid salt-extraction of high quality genomic DNA for PCR-based techniques. *Nucleic Acids Res.* **25**, 4692–4693 (1997).

69. B. Bushnell, BBTools software package (2014).

70. H. Li, B. Handsaker, A. Wysoker, T. Fennell, J. Ruan, N. Homer, G. Marth, G. Abecasis, R. Durbin; 1000 Genome Project Data Processing Subgroup, The sequence alignment/map format and SAMtools. *Bioinformatics* **25**, 2078–2079 (2009).

71. J. T. Page, Z. S. Liechty, M. D. Huynh, J. A. Udall, BamBam: Genome sequence analysis tools for biologists. *BMC Res. Notes* **7**, 829 (2014).

72. B. Q. Minh, H. A. Schmidt, O. Chernomor, D. Schrempf, M. D. Woodhams, A. von Haeseler, R. Lanfear, IQ-TREE 2: New models and efficient methods for phylogenetic inference in the genomic era. *Mol. Biol. Evol.* **37**, 1530–1534 (2020).

73. C. Zhang, M. Rabiee, E. Sayyari, S. Mirarab, ASTRAL-III: Polynomial time species tree reconstruction from partially resolved gene trees. *BMC Bioinform.* **19**, 153 (2018).

74. D. Bryant, R. Bouckaert, J. Felsenstein, N. A. Rosenberg, A. RoyChoudhury, Inferring species trees directly from biallelic genetic markers: Bypassing gene trees in a full coalescent analysis. *Mol. Biol. Evol.* **29**, 1917–1932 (2012).

75. R. Bouckaert, T. G. Vaughan, J. Barido-Sottani, S. Duchêne, M. Fourment, A. Gavryushkina, J. Heled, G. Jones, D. Kühnert, N. D. Maio, M. Matschiner, F. K. Mendes, N. F. Müller, H. A. Ogilvie, L. du Plessis, A. Popinga, A. Rambaut, D. Rasmussen, I. Siveroni, M. A. Suchard, C.-H. Wu, D. Xie, C. Zhang, T. Stadler, A. J. Drummond, BEAST 2.5: An advanced software platform for Bayesian evolutionary analysis. *PLOS Comput. Biol.* **15**, e1006650 (2019).

76. M. Malinsky, M. Matschiner, H. Svardal, Dsuite—Fast D-statistics and related admixture evidence from VCF files. *Mol. Ecol. Resour.* **21**, 584–595 (2021).

77. E. Garrison, G. Marth, Haplotype-based variant detection from short-read sequencing. arXiv:1207.3907 [q-bio.GN] (17 July 2012).

78. P. Danecek, A. Auton, G. Abecasis, C. A. Albers, E. Banks, M. A. DePristo, R. E. Handsaker, G. Lunter, G. T. Marth, S. T. Sherry, G. McVean, R. Durbin; 1000 Genomes Project Analysis Groupcorresponding author, The variant call format and VCFtools. *Bioinformatics* **27**, 2156–2158 (2011).

79. S. H. Martin, J. W. Davey, C. D. Jiggins, Evaluating the use of ABBA-BABA statistics to locate introgressed loci. *Mol. Biol. Evol.* **32**, 244–257 (2015).

80. K. L. Korunes, K. Samuk, Pixy: Unbiased estimation of nucleotide diversity and divergence in the presence of missing data. *Mol. Ecol. Resour.* **21**, 1359–1368 (2021).

81. S. Wang, E. Meyer, J. K. McKay, M. V. Matz, 2b-RAD: A simple and flexible method for genome-wide genotyping. *Nat. Methods* **9**, 808–810 (2012).

82. C. C. Chang, C. C. Chow, L. C. Tellier, S. Vattikuti, S. M. Purcell, J. J. Lee, Second-generation PLINK: Rising to the challenge of larger and richer datasets. *Gigascience* **4**, 7 (2015).

83. P. Danecek, J. K. Bonfield, J. Liddle, J. Marshall, V. Ohan, M. O. Pollard, A. Whitwham, T. Keane, S. A. McCarthy, R. M. Davies, H. Li, Twelve years of SAMtools and BCFtools. *Gigascience* **10**, giab008 (2021).

84. T. Rausch, T. Zichner, A. Schlattl, A. M. Stütz, V. Benes, J. O. Korbel, DELLY: Structural variant discovery by integrated paired-end and split-read analysis. *Bioinformatics* **28**, i333–i339 (2012).

85. V. Ficarrotta, J. J. Hanly, L. S. Loh, C. M. Francescutti, A. Ren, K. Tunström, C. W. Wheat, A. H. Porter, B. A. Counterman, A. Martin, A genetic switch for male UV iridescence in an incipient species pair of sulphur butterflies. *Proc. Natl. Acad. Sci. U.S.A.* **119**, e2109255118 (2022).

86. X. Messeguer, R. Escudero, D. Farré, O. Núñez, J. Martínez, M. M. Albà, PROMO: Detection of known transcription regulatory elements using species-tailored searches. *Bioinformatics* **18**, 333–334 (2002).

87. D. Farré, R. Roset, M. Huerta, J. E. Adsuara, L. Roselló, M. M. Albà, X. Messeguer, Identification of patterns in biological sequences at the ALGGEN server: PROMO and MALGEN. *Nucleic Acids Res.* **31**, 3651–3653 (2003).

88. B. Wang, A. H. Porter, An AFLP-based interspecific linkage map of sympatric, hybridizing Colias butterflies. *Genetics.* **168**, 215–225 (2004).

89. K. Maeki, C. L. Remington, Studies of the chromosomes of North American Rhopalocera. *J. Lepidopterists Soc.* **14**, 127–147 (1960).

90. P. Rastas, Lep-MAP3: Robust linkage mapping even for low-coverage whole genome sequencing data. *Bioinformatics* **33**, 3726–3732 (2017).

91. H. Li, Aligning sequence reads, clone sequences and assembly contigs with BWA-MEM. arXiv:1303.3997 [q-bio.GN] (16 March 2013).

92. V. Ahola, R. Lehtonen, P. Somervuo, L. Salmela, P. Koskinen, P. Rastas, N. Välimäki, L. Paulin, J. Kvist, N. Wahlberg, J. Tanskanen, E. A. Hornett, L. C. Ferguson, S. Luo, Z. Cao, M. A. de Jong, A. Duplouy, O.-P. Smolander, H. Vogel, R. C. McCoy, K. Qian, W. S. Chong, Q. Zhang, F. Ahmad, J. K. Haukka, A. Joshi, J. Salojärvi, C. W. Wheat, E. Grosse-Wilde, D. Hughes, R. Katainen, E. Pitkänen, J. Ylinen, R. M. Waterhouse, M. Turunen, A.

Vähärautio, S. P. Ojanen, A. H. Schulman, M. Taipale, D. Lawson, E. Ukkonen, V. Mäkinen, M. R. Goldsmith, L. Holm, P. Auvinen, M. J. Frilander, I. Hanski, The Glanville fritillary genome retains an ancient karyotype and reveals selective chromosomal fusions in Lepidoptera. *Nat. Commun.* **5**, 4737 (2014).

93. C. Trapnell, B. A. Williams, G. Pertea, A. Mortazavi, G. Kwan, M. J. van Baren, S. L. Salzberg, B. J. Wold, L. Pachter, Transcript assembly and quantification by RNA-Seq reveals unannotated transcripts and isoform switching during cell differentiation. *Nat. Biotechnol.* **28**, 511–515 (2010).

94. M. L. Borowiec, AMAS: A fast tool for alignment manipulation and computing of summary statistics. *PeerJ* **4**, e1660 (2016).

95. S. A. Smith, M. J. Moore, J. W. Brown, Y. Yang, Analysis of phylogenomic datasets reveals conflict, concordance, and gene duplications with examples from animals and plants. *BMC Evol. Biol.* **15**, 150 (2015).

96. A. Rambaut, A. Drummond, FigTree version 1.4.0 (2012).

97. A. Untergasser, I. Cutcutache, T. Koressaar, J. Ye, B. C. Faircloth, M. Remm, S. G. Rozen, Primer3—New capabilities and interfaces. *Nucleic Acids Res.* **40**, e115 (2012).

98. A. V. Z. Brower, A. V. L. Freitas, M.-M. Lee, K. L. Silva-Brandão, A. Whinnett, K. R. Willmott, Phylogenetic relationships among the Ithomiini (Lepidoptera: Nymphalidae) inferred from one mitochondrial and two nuclear gene regions. *Syst. Entomol.* **31**, 288–301 (2006).

99. B. S. Pedersen, R. L. Collins, M. E. Talkowski, A. R. Quinlan, Indexcov: Fast coverage quality control for whole-genome sequencing. *Gigascience* **6**, 1–6 (2017).

100. K. Kunte, W. Zhang, A. Tenger-Trolander, D. H. Palmer, A. Martin, R. D. Reed, S. P. Mullen, M. R. Kronforst, *doublesex* is a mimicry supergene. *Nature* **507**, 229–232 (2014).

101. F. Rodriguez-Caro, J. Fenner, S. Bhardwaj, J. Cole, C. Benson, A. M. Colombara, R. Papa, M. W. Brown, A. Martin, R. C. Range, B. A. Counterman, Novel *Doublesex* duplication associated with sexually dimorphic development of Dogface butterfly wings. *Mol. Biol. Evol.* **38**, 5021–5033 (2021).

102. R. Deshmukh, D. Lakhe, K. Kunte, Tissue-specific developmental regulation and isoform usage underlie the role of *doublesex* in sex differentiation and mimicry in *Papilio* swallowtails. *R. Soc. Open Sci.* **7**, 200792 (2020).

103. J. Hill, P. Rastas, E. A. Hornett, R. Neethiraj, N. Clark, N. Morehouse, M. de la Paz Celorio-Mancera, J. C. Cols, H. Dircksen, C. Meslin, N. Keehnen, P. Pruisscher, K. Sikkink, M. Vives, H. Vogel, C. Wiklund, A. Woronik, C. L. Boggs, S. Nylin, C. W. Wheat, Unprecedented reorganization of holocentric chromosomes provides insights into the enigma of lepidopteran chromosome evolution. *Sci. Adv.* **5**, eaau3648 (2019).
