# Supplementary material for: Evidence for a single, ancient origin of a genus-wide alternative life history strategy
Source: Sci Adv. 2023 Mar 22;9(12):eabq3713. doi: 10.1126/sciadv.abq3713 (PMC10032607; doi:10.1126/sciadv.abq3713)
Supplement: Supplementary file 3 — ST_covstat [file sciadv.abq3713_st_covstat.zip › ST_covstat/relative_read_depth/out-indexcov-roc-Sc0000103_pilon.html]

---

back to index
